# Restoration of mesenchymal retinal pigmented epithelial cells by TGFβ pathway inhibitors: implications for age-related macular degeneration

**DOI:** 10.1186/s13073-015-0183-x

**Published:** 2015-06-19

**Authors:** Monte J. Radeke, Carolyn M. Radeke, Ying-Hsuan Shih, Jane Hu, Dean Bok, Lincoln V. Johnson, Pete J. Coffey

**Affiliations:** Neuroscience Research Institute, University of California, Santa Barbara, CA USA; Departments of Ophthalmology and Neurobiology, Jules Stein Eye & Brain Research Institutes, David Geffen School of Medicine, University of California, Los Angeles, CA USA

## Abstract

**Background:**

Age-related macular degeneration (AMD) is a leading cause of blindness. Most vision loss occurs following the transition from a disease of deposit formation and inflammation to a disease of neovascular fibrosis and/or cell death. Here, we investigate how repeated wound stimulus leads to seminal changes in gene expression and the onset of a perpetual state of stimulus-independent wound response in retinal pigmented epithelial (RPE) cells, a cell-type central to the etiology of AMD.

**Methods:**

Transcriptome wide expression profiles of human fetal RPE cell cultures as a function of passage and time post-plating were determined using Agilent 44 K whole genome microarrays and RNA-Seq. Using a systems level analysis, differentially expressed genes and pathways of interest were identified and their role in the establishment of a persistent mesenchymal state was assessed using pharmacological-based experiments.

**Results:**

Using a human fetal RPE cell culture model that considers monolayer disruption and subconfluent culture as a proxy for wound stimulus, we show that prolonged wound stimulus leads to terminal acquisition of a mesenchymal phenotype post-confluence and altered expression of more than 40 % of the transcriptome. In contrast, at subconfluence fewer than 5 % of expressed transcripts have two-fold or greater expression differences after repeated passage. Protein-protein and pathway interaction analysis of the genes with passage-dependent expression levels in subconfluent cultures reveals a 158-node interactome comprised of two interconnected modules with functions pertaining to wound response and cell division. Among the wound response genes are the TGFβ pathway activators: *TGFB1*, *TGFB2*, *INHBA*, *INHBB*, *GDF6*, *CTGF*, and *THBS1*. Significantly, inhibition of TGFBR1/ACVR1B mediated signaling using receptor kinase inhibitors both forestalls and largely reverses the passage-dependent loss of epithelial potential; thus extending the effective lifespan by at least four passages. Moreover, a disproportionate number of RPE wound response genes have altered expression in neovascular and geographic AMD, including key members of the TGFβ pathway.

**Conclusions:**

In RPE cells the switch to a persistent mesenchymal state following prolonged wound stimulus is driven by lasting activation of the TGFβ pathway. Targeted inhibition of TGFβ signaling may be an effective approach towards retarding AMD progression and producing RPE cells in quantity for research and cell-based therapies.

**Electronic supplementary material:**

The online version of this article (doi:10.1186/s13073-015-0183-x) contains supplementary material, which is available to authorized users.

## Background

Wound responses encompass a wide range of processes directed at repairing tissue and cell damage. *In vivo*, wound responses are often secondary or bystander responses that occur as a result of damage to neighboring cells or tissue, however they can also occur at the single-cell level [1]. The source of damage can be physical trauma, microbial or viral infection, exposure to environmental toxins, genetically based dysfunction, or degenerative disease. The nature of the trauma can be acute or chronic. Some of the signals that can initiate wound responses are disruption of cell-cell contacts, loss of cell-substrate contacts, and exposure to blood components. In addition, innate inflammatory responses play a critical role in both the activation and orchestration of a wound response [2]. Wound response inactivation requires cessation of damage, wound closure, resolution of the inflammatory response, and tissue remodeling or regeneration. When the precipitating insult or injury is sustained, a chronic aberrant wound response can ensue and result in progressive disease; as is the case in chronic obstructive pulmonary disease or hepatitis-associated liver fibrosis [3, 4]. Although the core processes of wound repair are common to all tissues, there can be substantial differences depending on the extent and nature of the insult, as well as the affected tissues and cell types.

Fibrosis and uncontrolled neovascularization are components of several blinding disorders [5]. One of those diseases is age-related macular degeneration (AMD), a progressive disorder affecting the central region of the retina-choroid tissue complex. It is the leading cause of blindness for Caucasians in the United States [6], with 6.5 % of the total population aged 40 years and older having symptoms of AMD to some degree [7]. Dysfunction of the retinal pigmented epithelium (RPE) is generally considered to be central to the etiology of AMD. The RPE is a simple cuboidal monolayer that separates the neural retina from the highly vascular choroid. In addition to regulating the transport of metabolites and catabolites between the vascular system and the neural retina, the RPE performs a number of unique functions essential for the visual process and the maintenance of the photoreceptors.

The first clinically recognized signs of AMD are the accumulation of macular extracellular deposits, named drusen, located between the RPE and Brüch’s membrane, a multilayer extracellular matrix separating the RPE from the choroid. As the disease progresses the number and size of the drusen increase, RPE cells associated with drusen can lose their epithelial morphology, and there can be focal decreases or increases in RPE-associated pigment. During these initial stages of the disease, classified as early AMD [8–10], there is minimal perceptible loss of vision. Prominent loss of vision is only associated with late or advanced AMD, which is characterized by macular RPE atrophy with associated neural retina atrophy and/or fibrotic subretinal choroidal neovascularization. The former is referred to as geographic atrophy (GA) and comprises 8 % of the AMD population. The latter is classified as exudative or choroidal neovascular AMD (CNV) which affects 5 % of those with AMD [7].

The transition from early to advanced AMD has features consistent with the onset of an aberrant wound response resulting from an underlying degenerative disease and chronic inflammation. Drusen disrupt the normal tissue architecture, disrupt function by impeding transport between the RPE and choroid, and are sites of activation of the complement system [11, 12]. While the principal cause(s) of AMD that results in accumulation of drusen is debated, upwards of 75 % of AMD cases are associated with polymorphisms in components of the complement cascade [13], and cellular immune responses have been shown to be part of the AMD gene expression signature common to early AMD, GA, and CNV [14].

Here we report findings directed at furthering our understanding of the molecular basis of RPE wound response, its likely impact on RPE function, and possible role in AMD. Using a cell culture model system that considers prevention of monolayer formation as a surrogate for persistent wounding, coupled with a systems biology founded transcriptome analysis, we identify changes in expression in a number of genes encoding products that play a role in wound response or the cell cycle. Furthermore, using a pharmacological approach we determine that prolonged wound stimulus results in permanent activation of the TGFβ pathway that is independent of further wound stimulus. Finally, we investigate the possible role for wound responses in AMD by comparing the changes in gene expression in this cell culture model to previously reported AMD-associated gene expression changes.

## Methods

### Fetal RPE culture

RPE cells were isolated from fetal donor eyes (Advanced Biosciences Resources, Alameda, CA, USA) according to the methods of Hu and Bok [15, 16]. Beyond the initial isolation, all cell culture was carried out using a base medium described by Maminishkis *et al.* [17]. For 2–3 days post-plating the medium included 15 % heat inactivated fetal calf serum. Thereafter, it was reduced to 5 %. To generate a working cell bank, primary fetal RPE stocks were expanded approximately ten-fold and these working stocks were designated Passage 0 (P0). For routine serial passage, cells were harvested using trypsin digestion and plated at 4,000 cells/cm^2^. At approximately 80 % confluence (every 3–5 days depending on passage number) the cells were enzymatically harvested and re-seeded at 4,000 cells/cm^2^. Cultures were carried out on laminin-coated porous supports (mouse laminin, Life Technologies, Grand Island, NY, USA; Millicell-HA Culture Inserts, EMD Millipore Inc., Billerica, MA, USA) or laminin-coated tissue culture plastic as indicated. All cultures were fed every 2–4 days by complete exchange of the medium.

The human tissue used in this study was obtained by Advanced Biosciences Resources, (Alameda, CA, USA) with informed consent and in accordance with the World Medical Association Declaration of Helsinki and local legislation. The collection of the tissue by Advanced Bioscience Resources was evaluated and approved by the Western Institutional Review Board. No information relating to the identity of the donors was provided by Advanced Biosciences Resources.

### Microarray analysis

Total RNA was purified using miRNeasy mini-preps (Qiagen Inc., Valencia, CA, USA) and transcriptome profiles were determined using the Agilent Whole Human Genome 4 × 44 K *in situ* oligonucleotide platform (G4112F, Agilent Technologies, Inc., Santa Clara, CA, USA) and a two-color experimental design according to the methods of the manufacturer. After global background subtraction and Lowess normalization to correct for non-linear dye effects, the net intensities were determined by subtraction of the average value for the negative control probes. For the 200 probes with 10 replicates the average intensity was determined and the entire dataset was then quantile normalized. Detailed microarray methods, sample details, and data can be accessed through the Gene Expression Omnibus (GEO: GSE67899). Prior to statistical or bioinformatics analysis, probes that did not correspond to a known gene (as defined by an assignment of a HUGO gene symbol) and probes with an average signal intensity less than twice background in all samples were discarded. For those genes represented by multiple unique probes, the probe with the highest average intensity was selected.

### RNA-Seq analysis

Poly(A) + RNA was purified from 1 μg of total RNA using the Magnetic mRNA Isolation Kit (New England Biolabs, Inc., Ipswich, MA, USA) and then used to generate RNA-Seq libraries using the Ion Total RNA-Seq Kit V2 (Life Technologies, Inc., Grand Island, NY, USA). The resulting libraries were sequenced on Ion PGM or Ion Proton next-generation sequencers. Sequence results were aligned to the human transcriptome and genome (hg38) using a two-stage pipeline employing TopHat2 [18] and TMAP (Life Technologies, Inc.) read aligners. The number of reads per protein coding mRNA was determined using Partek Genomics Suite (Partek Inc., St. Louis, MO, USA) and the dataset was normalized using the trimmed mean of the M-values method [19]. Genes with read counts per million (RPM) ≥1 in three or more samples were selected and differential expression and statistical analysis was carried out using edgeR [20, 21]. The RNA-Seq data and detailed methods can be accessed through the Gene Expression Omnibus (GEO: GSE67899).

### Bioinformatics analysis

Cluster analysis was carried out on range centered log transformed data using AutoSOME 2.1 (500 ensemble runs, *P* = 0.03, unit variance, sum of squares = 1, and precision settings) [22]. Individual clusters were assigned to general groups following visual examination of the expression patterns. Gene ontology enrichment analysis was performed using David 6.7 and default settings [23, 24]. Enrichment for RPE signature genes was carried out manually using the combined RPE gene lists reported by Strunnikova *et al.* [25] and Booij *et al.* [26]. Interaction analysis was accomplished using STRING 9.1 [27] using a medium confidence setting and the Experiment and Databases prediction methods. The resulting network was manually rendered using Cytoscape 3.0.2 [28].

The assessment of the involvement of RPE wound response in AMD was carried out using the macular, extramacular, and combined macular-extramacular RPE-choroid and retina AMD gene list published by Newman *et al.* ([14], Additional file 1: Table S1). Redundant probes for the same gene in an AMD disease class were eliminated and AMD genes among the 13,791 genes with above background expression in P0 or P5 RPE cells were identified. AMD-Up genes expressed in RPE cells were cross-referenced with a list of 2,684 genes with increased expression in P5 RPE cells. RPE expressed AMD-Down genes were matched to the 2,856 genes with decreased expression following passage. Differential expression was defined as having a 1.5-fold or greater change in expression in all three donor cultures and a minimum average fold change of 2.0 as a function of passage in at least one of the four different time points examined. Enrichment odds ratios and *P* values were determined using Fisher’s exact test and the aforementioned values for the sizes of the P5-Up and P5-Down gene sets and the expressed RPE cell genome.

### Real-time quantitative PCR

Real-time quantitative PCR (RT-qPCR) was performed using PrimeTime® PCR Assays (Integrated DNA Technologies, Inc., Coralville, IA, USA). Assay information is provided in Additional file 1: Table S1. For each sample, cDNA was generated from total RNA using the iScript cDNA Synthesis Kit (Bio-Rad Laboratories, Inc., Hercules, CA, USA). For each gene of interest, RT-qPCR was carried out in duplicate using iTaq Universal Probes Supermix and the following thermal cycle profile: 95 °C for 1 min, 45 × (95 °C for 5 s, 60 °C for 1 min). Gene expression levels were calculated assuming a PCR efficiency of 100 % and normalized to the geometric mean of three housekeeping genes (RPL15, NDUFA11, and UBB) whose expression levels were found to be constant based on the microarray analysis and the PCR analysis methods of Vandesompele *et al.* [29].

### Immunocytochemistry

Cells cultured on microporous inserts were fixed with 4 % paraformaldehyde in phosphate buffered saline (PBS) for 10 min, after which they were transferred to 0.4 % paraformaldehyde in PBS and stored at 4 °C. In preparation for immunolabeling, portions of insert were excised, rinsed with PBS, and incubated in PBS containing 1 mg/mL bovine serum albumin and 0.1 % Triton X-100 (PBT) with 5 % normal donkey serum to block non-specific binding. Following a brief wash in PBT, the specimens were incubated overnight with primary antibody diluted in PBT. In those cases where unlabeled primary antibodies were used, the specimens were washed in PBT and incubated with fluorescently labeled secondary antibodies for 4 h. The specimens were then washed with PBT followed by staining with Alexa Flour 488 phalloidin and Hoescht 33342 to label actin filaments and DNA. Images of the labeled cells were collected using laser scanning confocal microscopy. The antibodies used in this study and their dilutions were: mouse anti-ACTA2, 1:500 (#MA5-15871: ThermoScientific, Waltham, MA, USA); rabbit anti-BEST1, 1:300 (#5466: EMD Millipore, Temecula, CA, USA); mouse anti-PMEL, 1:50 (#M0634: Dako, Carpenteria, CA, USA); Alexa Fluor 594 mouse anti-TJP1, 1:100 (#339194: Life Technologies); Alexa Fluor 546 donkey anti-mouse IgG, 1:200 (#A10036: Life Technologies); Alexa Fluor 647 donkey anti-mouse IgG(H + L), 1:200 (#A31571: Life Technologies); and Alexa Fluor 647 donkey anti-rabbit IgG(H + L), 1:200 (#A31573: Life Technologies).

## Results

### Prolonged subconfluent culture results in loss of the epithelial phenotype

**Fig. 1:**
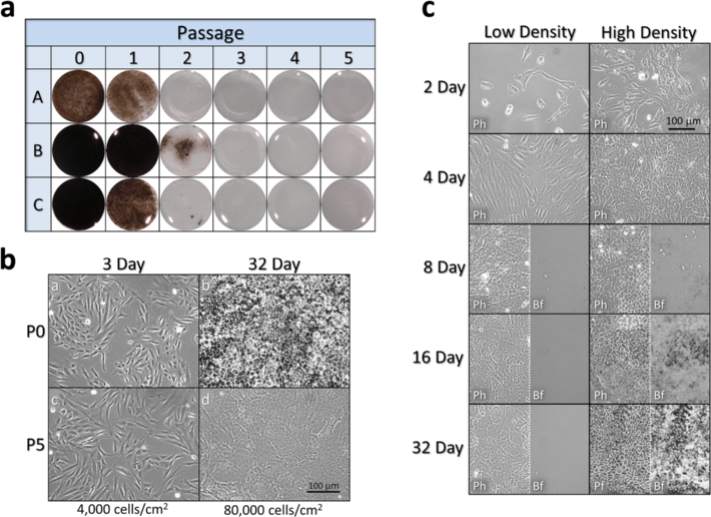
Sustained subconfluent culture results in the loss of capacity to differentiate. Fetal RPE cells were plated at 4,000 cells/cm^2^ and passaged at subconfluence every 3–5 days. At each passage a portion of the cells were plated at 80,000 cells/cm^2^ and maintained without further passaging. **a** Whole culture photographs of 64-day serially passaged RPE cells from three different donors (A, B, and C) plated on microporous inserts at 80,000 cells/cm^2^. **b** Phase contrast micrographs of 3-day low density cultures of P0 (a) and P5 (c) cells plated at low density reveal only minor differences in phenotype. At 32 days P0 cultures (b) plated at high density have a prototypical pigmented cobblestone RPE phenotype, while P5 cells (d) are not pigmented and have a disorganized morphology. **c** Passage 0 cells were plated at 4,000 and 80,000 cells/cm^2^ on tissue culture plastic and maintained without further passage. Phase contrast (Ph) and brightfield (Bf) micrographs of the cultures as function of time show that high density plating is a requirement for normal RPE differentiation

It has been appreciated for some time that primary RPE cells, like many cells, undergo a permanent switch in phenotype with repeated passage [30]. Minimally passaged cultured RPE cells have a non-epithelial morphology reminiscent of fibroblasts while they are subconfluent and mitotically active. It is only after they reach confluence that they attain an epithelial morphology and begin to accumulate pigment. The change in epithelial to fibroblast-like phenotype is commonly referred to as an epithelial-to-mesenchymal transition (EMT) and is a component of development and successful wound repair where the cells return to their normal differentiated state via a mesenchymal-to-epithelial transition. However, with repeated passage at sub-confluence there is a marked decrease in the capacity to pigment (Fig. 1a) and to acquire an epithelial morphology (Fig. 1b) post-confluence. In addition to a passage-dependent loss of potential to differentiate, minimally passaged RPE cells also fail to differentiate when seeded at low density (Fig. 1c). Repeated passage also results in a decreased rate of cell doubling, a lower final cell density, and loss of cells post-confluence (Additional file 2: Figure S1).

### Passage-induced changes in phenotype are accompanied by wholesale changes in gene expression

To gain insight into the molecular basis of the phenotypic switch, we carried out a whole transcriptome analysis of RPE as a function of passage and time in culture. Passage 0 (P0) and Passage 5 (P5) RPE cells from three different donors were plated at a density conducive to differentiation and maintained in culture for 16, 32, and 64 days. In addition, P0 and P5 cultures were seeded at low density and collected after 3 days while still subconfluent. Genome-wide transcriptome profiles were then determined by microarray analysis. Of the 19,595 non-redundant RefSeq genes represented on the microarrays, nearly 13,800 had detectable levels of expression as defined by net signal intensities greater than twice the estimated background. To identify genes that were differentially expressed, a fold change criteria was employed requiring each intra-donor pairwise comparison to have a fold change of ≥1.5 and the mean fold change for all donors to be ≥2.0. When this criterion was applied to all possible inter-donor comparisons within a condition it was determined that the number of differences expected by random chance ranged from 2 (3-day P0) to 77 (16-day P5).

**Fig. 2:**
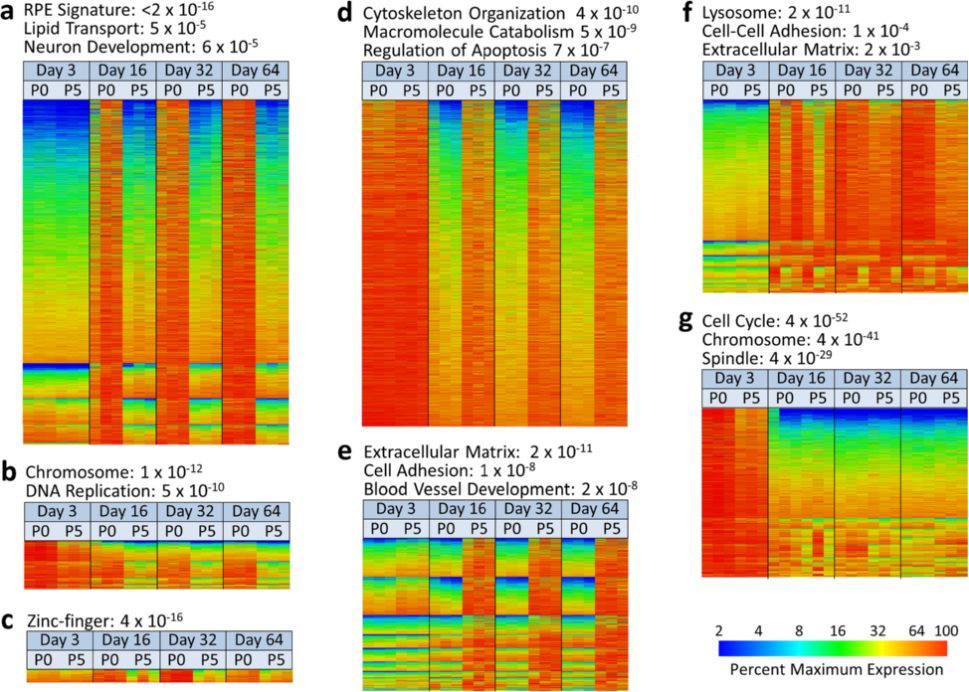
Genome-wide transcriptome profiling and cluster analysis uncovers functionally related groups of genes that are co-regulated as a function of passage and time. Genes with a two-fold or greater change in gene expression as a function of passage or time were segregated based on their expression profiles using AutoSOME 2.1. Individual clusters with similar expression profiles were placed in major expression groups (**a**-**g**) following manual examination. The headers of each group are representative of gene ontology classes that are over-represented in each group. The value associated with the ontology groups is the enrichment *P* value. The color scale indicates the expression level as a percentage of the maximum value for that gene. Each column represents a unique biological replicate (n = 3). Rows represent unique genes. The corresponding gene identifiers and associated data can be found in Additional file 3: Table S2

Over two-thirds of the genes with detectable signals had two-fold or greater differences in expression as a function of passage or time in culture. Nearly 5,500 genes had expression differences as a function of passage alone. To better categorize the differentially expressed genes they were arranged into groups based on their expression profiles using AutoSOME [22], an assumption-free clustering method (Fig. 2). Three major expression profile categories were identified. The first (Groups a-c) consists of genes that are preferentially expressed in differentiation-competent (P0) cells. The second category (Groups d and e) is comprised of genes that are most highly expressed in differentiation-incompetent (P5) cells. Group d is primarily comprised of genes that are expressed in subconfluent cells and fail to turn off in confluent P5 cells. Group e contains genes preferentially expressed in confluent P5 cells. The last category (Groups f and g) is made up of genes whose expression correlates with the state of confluence. A listing of the genes associated with these expression groups and the associated data is provided in Additional file 3: Table S2.

To help elucidate how these changes in gene expression might account for the switch to a persistent mesenchymal phenotype, the different cluster groups were subjected to gene ontology enrichment analysis [23, 24]. Consistent with current understanding of the RPE phenotype, Group a, which is comprised of genes most highly expressed in terminally differentiating RPE, is enriched for genes previously identified as RPE marker genes [25, 26] or with functions consistent with the differentiated RPE phenotype. Among the most regulated genes in this group are the pigmentation related genes *TYR*, *TYRP1*, *DCT*, and *OCA2*; *TRPM1* and *TRPM3* which harbor the RPE microRNAs MIR204 and MIR211 in their intronic regions [31]; and genes associated with the visual cycle (*LRAT*, *RPE65*, *RPLBP1*, *RDH5*, and *RBP1*). Conversely, Groups d and e, which consists of genes whose levels are most abundant in dedifferentiated RPE, are enriched for genes commonly associated with a mesenchymal phenotype or wound response such as *ACTA2*, *CDH2*, *CTGF*, *FN1*, *TGFB1*, and *TGFB2*. While Group f, is not statistically enriched for RPE signature genes, many of the genes in this group are critical for important RPE functions or development (*PMEL*, *AQP1*, *STRA6*, *BMP7*).

### Identification of a core set of genes associated with the potential to acquire a RPE phenotype

To gain further understanding into the molecular basis underlying the switch to a persistent mesenchymal state, attention was directed towards the subconfluent cells. Unlike the case with confluent P0 and P5 cells where there are enormous changes in gene expression and virtually every regulatory pathway of potential interest is impacted, only 565 genes are differentially expressed between subconfluent P0 and P5 cells. Approximately half of these transcripts are more abundant in P5 cells. Given that it is determined that the P0 cells will become RPE and the P5 cells will become mesenchymal, it follows that the key differences that dictate their final phenotype might lie in this relatively small set of genes. Gene ontology enrichment analysis of this gene subset demonstrates that it is highly enriched for genes associated with the cell cycle (n = 53, *P* value <10^−7^) and genes often associated with wound responses (extracellular matrix: n = 49, *P* value <10^−18^; cell adhesion: n = 50, *P* value <10^−7^; growth factor binding: n = 15, *P* value <10^−5^; actin binding: n = 26, *P* value <10^−5^; positive regulation of apoptosis: n = 29, *P* value <10^−3^).

**Fig. 3:**
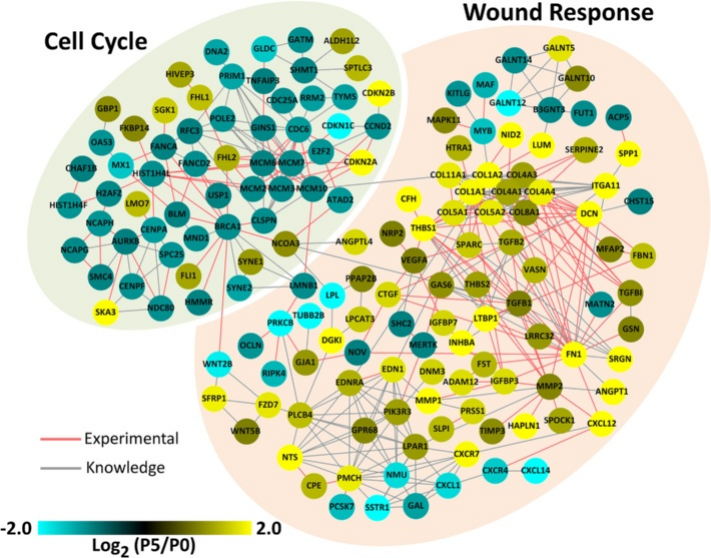
Differential expression of a core set of genes whose products comprise a functional interactome is an underlying feature of chronically wounded RPE cells. The 565 genes with two-fold or greater differences in expression between 3-day P0 and P5 cultures were analyzed using STRING 9.1 to identify genes whose products have experimentally determined protein-protein interactions (Experimental) or are part of a common curated pathway (Knowledge) and the resulting interactome was rendered using Cytoscape 3.0.2. The interactome is segregated into two sub-modules, one enriched for gene products involved in the cell cycle and the other for genes that are often associated with the response to wound stimuli. The line color indicates the nature of the connection with direct protein-protein interactions taking precedence over common pathway. The gene node color represents the log_2_ transformed P5:P0 gene expression ratio as determined by microarray analysis

To help determine how the products of this core set of differentially expressed genes might act to control the phenotypic fate, an interactome analysis was performed. As shown in Fig. 3, nearly one-third of the products of this core gene set could be organized into a network based on direct protein-protein interactions or being part of a common curated pathway. Moreover, this 158-node interactome could be segregated into two modules; one that is involved in the cell cycle and one that is enriched for proteins commonly associated with wound responses. In general, the cell cycle transcripts are found at higher levels in minimally passaged RPE and the wound response transcripts are more abundant in highly passaged RPE.

Interestingly, within the wound response module, over 70 % of the proteins are found in the extracellular compartment, consistent with a possible role in autocrine/paracrine signaling. Most notable in the wound response module are a group of highly interconnected nodes focused around the TGFβ family ligands TGFB1, TGFB2, and INHBA. Additional groups of potential interest are chemokines and chemokine receptors (CXCL1, CXCL12, CXCL14, CXCR4, CXCR7), WNT signaling (FZD7, SFRP1, WNT2B, WNT5B), regulators of angiogenesis (ANGPT1, ANGPTL4, VEGFA), insulin growth factor binding proteins (IGFBP3, IGFBP7), and G-protein coupled receptors and ligands (EDNR, LPAR1, SSTR1 and EDN1, GAL, NMU, NTS, PMCH). Gene products of note in the mitosis module are CCND2 and the cyclin-dependent kinase inhibitors CDKN1C, CDKN2A, and CDKN2B. Many of the remaining gene products in the cell cycle module are components of the replication machinery.

### Preservation and restoration of the RPE phenotype by wound response inhibitors and stimulation of mitosis

Based on the results of the transcriptome analysis we hypothesized that the negative effects of subconfluent culture on RPE differentiation might be prevented or reversed using inhibitors of the aforementioned wound response signaling pathways or FGF2 to stimulate cell division. As an initial test, P0 cells were plated at low density to inhibit terminal RPE differentiation, and maintained in medium supplemented with A-83-01, thiazovivin, LDN-193189, XAV939, or FGF2 for 32 days. A-83-01 blocks the ligand activated kinase activity of the type I TGFβ receptors ACVR1B, TGFBR1, and ACVR1C [32]. These receptor subunits, which are considered to be members of the TGFβ receptor subfamily, are also referred to as activin-like receptors (ALK) 4, 5, and 7. Ligands that have been reported to activate these receptor kinases include INHBA, INHBB, GDF11, TGFB1, and TGFB2 [33]. Based on the transcriptome analysis, only ACVR1B and TGFBR1 are expressed in differentiated or non-differentiated RPE cells. Thiazovivin is an inhibitor of the rho-associated, coiled-coil-containing protein kinases, ROCK1 and ROCK2 [34]. ROCK1 and ROCK2 regulate the actin cytoskeleton and participate in a number of relevant pathways including cadherin, chemokine, focal adhesion, WNT, and TGFβ signaling [35]. LDN-193189 is an inhibitor of the BMP subfamily of the TGFβ type I receptors [36, 37]. Members of this family with detectable microarray signals in RPE are ACVR1 (ALK2), BMPR1A (ALK3), and BMPR1B (ALK6). Among the ligands that activate these receptors are BMP2, BMP4, BMP7, GDF5, GDF6, INHBA, and INHBB [33]. XAV939 is an antagonist of WNT signaling that acts indirectly to increase the level of axin, a key protein involved in the degradation of β-catenin [38]. Basic FGF (FGF2) is a widely recognized mitogen that has been previously shown to stimulate RPE cell division [39, 40].

**Fig. 4:**
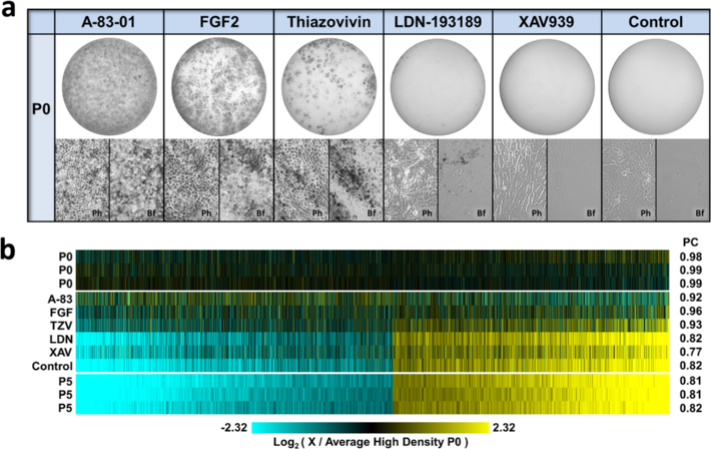
FGF2 or small molecule inhibitors of wound response promote differentiation at low plating density. Passage 0 cells were plated at 4,000 cells/cm^2^ and grown in the presence of 500 nM A-83-01, 2 ng/mL FGF2, 500 nM thiazovivin, 200 nM LDN-193189, or 5 μM XAV939. The vehicle (DMSO) alone served as the control. **a** Whole culture images (above), phase contrast (Ph) and brightfield micrographs (Bf) taken at day 32 demonstrate that A-83-01, FGF2, and thiazovivin treated cultures maintain the capacity to differentiate after low density plating. **b** Whole transcriptome profiles of the 32-day treated and control low density P0 cultures were determined and compared to the expression profiles of differentiated 32-day high density P0 cultures. The transcriptome profiles of 32-day differentiated (P0) and undifferentiated (P5) RPE cells plated at 80,000 cells/cm^2^ from three donors are shown for comparison. Only genes with two-fold or greater P0:P5 expression ratios and a P0 or P5 net intensity ≥15 were evaluated. The color scale indicates the log_2_ transformed expression level relative to the mean value of the 32-day high density P0 cells. Black indicates identity. The values on the right indicate the Pearson’s product–moment correlation coefficient (PC) for all expressed genes relative to the mean of the high density P0 profiles

As shown in Fig. 4a, all of the inhibitors and FGF2 have the capacity to improve the morphology of low density P0 RPE cultures, albeit to varying extent. The WNT signaling inhibitor, XAV939, and the BMP subfamily receptor inhibitor, LDN-193189, are the least effective. XAV939 treated cells have a more regular, somewhat epithelial-like morphology when compared to untreated control cells, however there is no evidence of pigmentation. LDN-193189 treatment has minimal effect on gross morphology, but there is improved pigmentation with foci of sparse pigmentation being evident throughout the culture. In contrast, inhibition of TGFβ subfamily receptor and ROCK activity using A-83-01 or thiazovivin, as well as treatment with the mitogen FGF2 are strikingly more effective at preventing the loss of phenotype. While there are quantitative differences, A-83-01, thiazovivin, and FGF2 treated cultures all have substantial regions of pigmentation and prototypical RPE morphology. Furthermore, when cells are plated at sufficient density to allow for differentiation in control medium, both A-83-01 and thiazovivin treatment result in more uniform phenotype (Additional file 2: Figure S2). No negative effects of LDN-193189 on gross morphology were apparent in high density cultures. On the other hand, XAV939 treated high density cultures had reduced levels of pigmentation and an apparent increase in cell size relative to control cultures.

In addition to assessing the effects of these agents on gross morphological criteria, their effects on global gene expression were evaluated (Fig. 4b). In general, the effects of the treatments on gene expression mirrored that observed with respect to morphology and pigmentation. Cells treated with A-83-01, FGF2, and thiazovivin have transcriptome profiles most similar to high density P0 cultures. Interestingly, A-83-01 treatment results in a significant number of RPE marker genes with elevated expression (Fisher’s exact test, *P* value <0.001) and genes associated with P5 cells with reduced expression (Fisher’s exact test, *P* value <10^−5^) relative to untreated P0 cells; suggesting that there may be some degree of active TGFβ signaling in P0 cells.

**Fig. 5:**
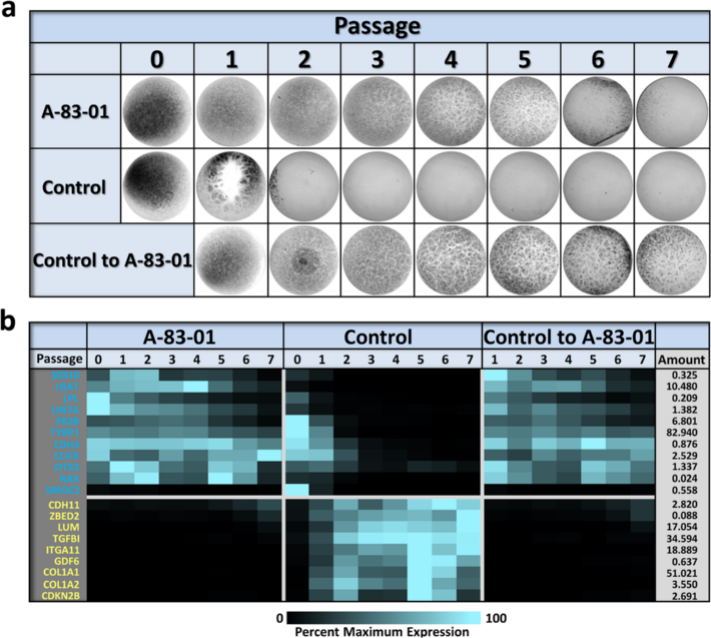
Inhibition of TGFBR1/ACVR1 signaling extends the effective lifespan of RPE cells. Cells were serially passaged using medium supplemented with 500 nM A-83-01 or normal medium. At each passage, parallel cultures were established using a plating density of 80,000 cells/cm^2^ and maintained for 32 days in the medium used for passage. To determine whether A-83-01 could restore the capacity to differentiate after passage in control medium, cells passaged in control medium were also plated into medium supplemented with 500 nM A-83-01. **a** Whole culture photographs of the high density cultures taken after 32 days (A-83-01, cells passaged and maintained in A-83-01; Control, cells passaged and maintained in normal medium; Control to A-83-01, cells passaged in normal medium and switched to A-83-01 at the indicated passage). See Additional file 2: Figure S3 for phase contrast and brightfield micrographs. **b** Analysis of RNA expression of select markers of RPE differentiation (blue gene symbols) and RPE wound response (yellow gene symbols) using RT-qPCR. The color scale indicates the expression level relative to the maximum value for that gene. The expression level for the maximum value relative to the housekeeping genes is listed to the right of the heatmap (Amount)

With the finding that inhibition of TGFβ receptor signaling supported RPE differentiation at low plating density, further investigations were directed at assessing if A-83-01 could prevent the loss of, or even rescue the RPE phenotype after repeated passage (Fig. 5). To determine if TGFβ pathway inhibition would block the loss of phenotype, cells were passaged at subconfluence in the presence of A-83-01. To ascertain whether RPE cells could be passaged in normal medium and subsequently rescued by A-83-01, cells were passaged in normal medium and at each passage a portion of the culture was seeded at high density in medium containing A-83-01. Based on the degree of pigmentation, cells cultured in the continual presence of A-83-01 continue to differentiate for at least five passages; four passages beyond that observed in the absence of A-83-01. The ability of A-83-01 to rescue the cells was even more pronounced. Cells grown in normal medium and subsequently treated with A-83-01 had substantial levels of pigmentation even after seven preceding passages in control medium. Consistent with the results obtained at low density plating (see Fig. 4a), A-83-01 also promoted the establishment of the prototypical epithelial morphology in this experimental paradigm (Additional file 2: Figure S3). In addition to its effects on gross morphology, immunological and cytological examination reveals that A-83-01 suppresses the expression of smooth muscle actin (ACTA2), preserves the prototypical cortical actin cytoskeleton and co-localization of tight junction protein 1, and maintains the expression and localization of the RPE marker proteins, PMEL and BEST1 (Additional file 2: Figure S4). While the beneficial effects of inhibition of TGFBR1 and ACVR1 signaling are readily apparent, it should be noted that the effects are not limitless. Eventually the cells fail to differentiate, suggesting there may be more to the loss of capacity to differentiate with increasing passage than TGFβ-mediated mesenchymal transition alone.

The effect of A-83-01 on the expression of a set of representative passage-dependent genes was evaluated using RT-qPCR (Fig. 5b and Additional file 2: Figure S5). In general, A-83-01 treatment promoted the expression of genes that are preferentially expressed in differentiated RPE (RPE genes) and suppressed the expression of genes that are most highly expressed in non-differentiated RPE (Wound Response genes). The one exception was *SMOC2*, which is normally expressed in differentiated P0 and P1 cells maintained in control medium. Not only is the expression of SMOC2 not maintained by A-83-01, it is inhibited by A-83-01. As was seen in the low density plating paradigm (see Fig. 4), P0 cells treated with A-83-01 and plated at high density have higher than normal levels of some RPE transcripts (LIN7A, LPL, OTX2 and RAX) and lower than normal levels of some Wound Response transcripts (COL1A1, COL1A2, GDF6, ITGA11, TGFBI) (Additional file 2: Figure S6).

While in general A-83-01 promotes the expression of RPE marker genes and suppresses the expression of passage-induced genes, there is evidence of finer patterns of expression that are not always in direct relationship with the gross phenotype (Additional file 2: Figure S5). For example, in the case of the RPE genes, the level of CDH3, CLIC6 and TYRP1 transcripts remain relatively constant in the presence of A-83-01 at all passages. Whereas, LIN7A, LPL, LRAT and SOX10 levels decrease significantly with passage, even in the presence of A-83-01. In the case of the genes that are most highly expressed in non-differentiated RPE and are suppressed by A-83-01, three genes (*LUM*, *CDH11*, and *ZBED2*) have evident increases in expression at the later passages when there is a notable loss of capacity to terminally differentiate; while the expression of the remaining wound response genes remained blocked by A-83-01. Some genes (*OTX2*, *RAX*, *CDKN2B*, *COL1A1*, and *COL1A2*) even have apparent cyclical expression profiles as a function of passage.

**Fig. 6:**
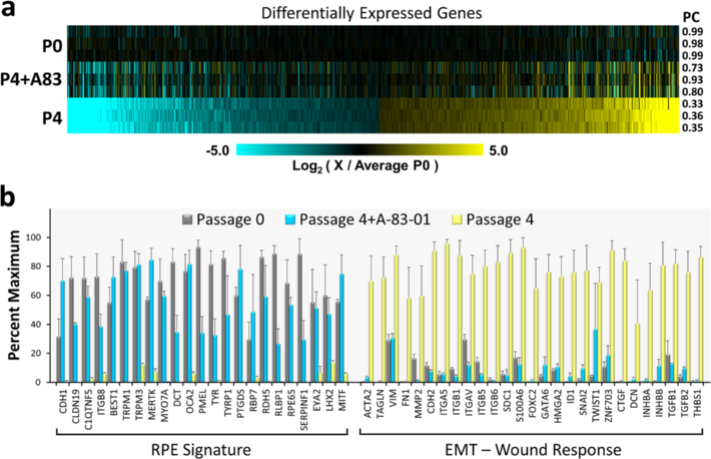
A-83-01 treatment largely restores the RPE transcriptome. Untreated P0 cells, untreated P4 and P4 cells treated with 500 nM A-83-01 were seeded at 80,000 cells/cm^2^. After 32 days the cells were harvested and their transcriptome profiles were determined using RNA-Seq. **a** Heatmap showing the expression levels relative to the mean of the three P0 cultures. Only the 4,590 genes with significant (FDR ≤0.01) expression differences between P0 and untreated P4 cells are depicted. The values on the right indicate the Pearson’s product–moment correlation coefficient (PC) for all expressed genes relative to the mean of the high density P0 profiles. **b** Relative expression levels of select genes with well-known roles in RPE-specific functions or EMT and wound response. For each gene, the expression levels are normalized relative to the maximum value (mean +/− SEM, n = 3)

As a broader assessment of the efficacy of A-83-01 to rescue the RPE phenotype, we also carried out a comparative RNA-Seq based transcriptome analysis of differentiated P0 RPE, mesenchymal P4 cells, and A-83-01 rescued P4 cells. Consistent with the microarray analysis, 4,590 genes were found to be differentially expressed (FDR ≤0.01) between 32 day old differentiated P0 and untreated P4 cells cultures with the genes being split roughly equally between being upregulated or downregulated. Seventy-five percent of the genes found to be differentially expressed using RNA-Seq were also identified by microarray analysis. When the expression level of these passage-dependent genes were evaluated in cells that had been passaged four times in normal medium and then treated with A-83-01, 87 % had at least a 25 % recovery in expression relative to P0 cells (Fig. 6a). Over 75 % of the passage-dependent genes had a recovery of 50 % or better following A-83-01 treatment. As highlighted in Fig. 6b, genes whose expression is enhanced by A-83-01 include those with well know roles in key RPE functions such as adhesion (*CHD1*, *CLDN19*, *C1QTNF5*, *ITGB8*), ion transport (*BEST1*, *TRPM1*, *TRPM3*), rod outer segment phagocytosis (*MERTK*, *MYO7A*), pigmentation (*DCT*, *OCA2*, *PMEL*, *TYR*, *TYRP1*), retinoid metabolism (*RBP7*, *RDH5*, *RLBP1*, *RPE65*), pigment epithelial growth factor (*SERPINF1*), and RPE gene regulation (*EYA2*, *LHX2*, *MITF*). As would be predicted, A-83-01 treatment downregulates the expression of well recognized markers or regulators of EMT and TGFβ signaling to levels similar to or below that seen in differentiated P0 RPE.

Finally, to address whether the effects of A-83-01 on RPE phenotype were specific to inhibition of the TGFβ pathway, dose–response analyses of A-83-01 and two additional TGFBR1/ACVR1B inhibitors, RepSox [41–43] and SB-431542 [44, 45], were performed (Additional file 2: Figure S7). A-83-01, RepSox, and SB-431542 all promoted RPE differentiation and gene expression, and blocked the expression of wound response genes in a typical dose-responsive manner. Moreover, based on the subset of genes examined by quantitative PCR, treatment with RepSox resulted in an expression profile most similar to that of differentiated P0 cells. Like A-83-01, both RepSox and SB-431542 can increase the number of passages over which RPE can differentiate and reverse the effects of passage (data not shown).

### Altered gene expression of TGFβ pathway components

**Fig. 7:**
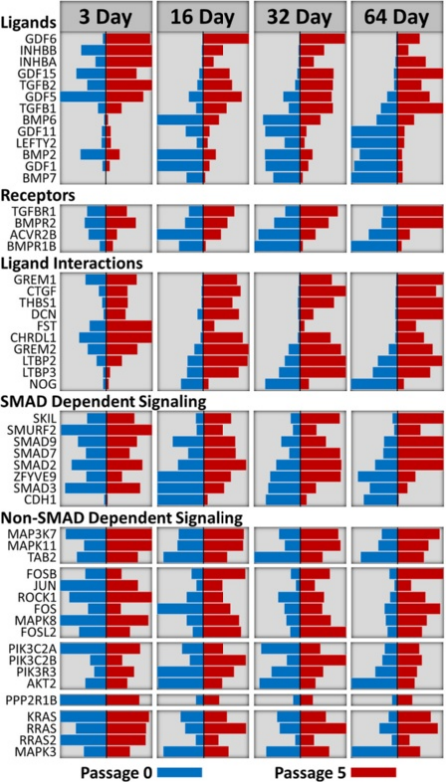
Sustained subconfluent culture results in widespread changes in the expression of genes involved in TGFβ signaling. One hundred and fourteen genes whose products are involved in TGFβ signaling were identified following a survey of curated pathways and the literature. Shown are those genes with a two-fold or greater difference in expression as a function of passage and a *P* value ≤0.05 (one-way ANOVA, n = 3) at any of the four time points, as determined by microarray analysis. The length of each bar indicates the level of expression relative to the maximum expression value for that gene. The axis of the P0 (blue) and P5 (red) bars are inverted to allow for ease of comparison

To gain further insight into how changes in gene expression might account for the loss of capacity to differentiate, known participants in TGFβ signaling with passage-dependent changes in expression were identified. Of the 114 TGFβ signaling genes with detectable expression examined, 53 had two-fold or greater differences in expression between P0 and P5 cells (one-way ANOVA *P* value ≤0.05) in at least one of the four measured time points (Fig. 7). Taking into consideration that A-83-01 inhibits signaling via TGFBR1 and ACVR1B, the pattern and extent of changes in expression, and their documented function, two groups of genes of interest were identified. The first group is comprised of the ligands INHBA, INHBB, TGFB1, and TGFB2. These ligands all signal via the appropriate receptors and are found at elevated levels in both subconfluent and confluent late-passage cells. The second group is comprised of genes coding for proteins that regulate the activity and/or availability of TGFβ ligands. Within that group, *THBS1*, *CTGF*, and *DCN* are of most interest. All three of these genes are found at substantially higher levels in subconfluent and confluent P5 cells, and the products of these genes promote the activity of TGFB1 and TGFB2. Thrombospondin-1 is capable of directly activating latent TGFβs by disrupting the interaction with the latent TGFβ binding proteins [46–48]. CTGF directly binds both TGFβs and THSB1, which then results in enhanced signaling through an ill-defined mechanism [49, 50]. Although DCN is most often associated with inhibition of TGFβ signaling [51], this is not always the case. In proliferating myoblasts DCN promotes the action of TGFβs [52–55]. One other ligand of note is GDF6. Its expression is almost exclusive to P5 cells and as such it is a prime candidate for participating in the prevention of RPE differentiation. Of note, the levels of expression of these regulators of the TGFβ pathway in passaged RPE treated with A-83-01 are the same or lower than that of differentiated P0 RPE (see Figs. 5 and 6).

### Altered expression of RPE wound response genes in AMD

**Fig. 8:**
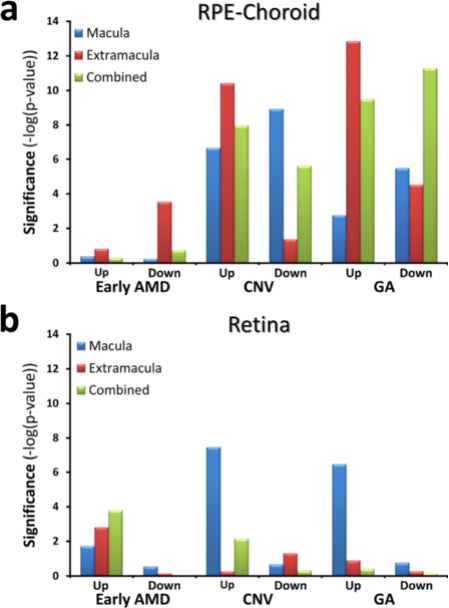
Genes associated with the RPE wound response are over-represented among those genes with altered expression in advanced AMD. Genes with two-fold increased or decreased expression as a function of passage were cross-referenced with the Up- and Down- RPE-choroid and retina AMD-associated gene sets reported by Newman *et al.* [14]. The charts depict the significance (−log(*P* value), Fishers’s exact test) of the overlap relative to the null hypothesis for the RPE-choroid (**a**) and retina (**b**) gene sets. Combined refers to the gene set resulting from the pooled analysis of the macular and extramacular samples

Consistent with a role of wound response in the progression of AMD, we have previously shown that AMD is associated with elevated levels of TGFB2 mRNA in both the RPE-choroid and retina [14]. To further evaluate the role of wound response in AMD we compared the list of passage-dependent genes reported here with our list of early AMD, CNV, and GA candidate genes. Only genes expressed by cultured RPE cells were considered, and for a match to be recorded the direction of change had to be consistent with the hypothesis that wound response is a component of AMD. As highlighted in Fig. 8, there is a highly significant enrichment of genes that change as a function of passage in CNV and GA, in both the RPE-choroid and retina. In the RPE-choroid, the enrichments were seen in both the macular and the extramacular regions, for both the upregulated and downregulated genes. In the retina, the overlap was largely limited to the macula and was only observed among the genes with increased expression in AMD and passaged RPE. The lack of enrichment with the downregulated class in the retina is expected as most of the genes with decreased expression in the cell culture model are associated with RPE-specific function. Consistent with the lack of overlap between genes that change in early AMD and passage, we found no significant overlap with the sets of AMD genes reported by Whitmore *et al.* [56] or Hunter *et al.* [57] whose analysis was limited to early AMD or AMD in general.

### Altered expression of AMD-associated genes as a function of prolonged wound stimulus

**Fig. 9:**
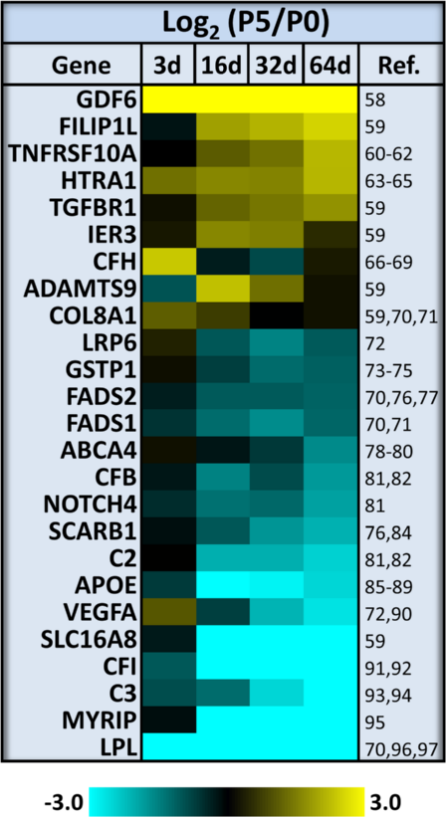
AMD risk-associated genes with altered expression in late-passage RPE. Of 63 genes with reported association with altered risk for AMD that were evaluated, 50 were found to be expressed by cultured RPE. Of the expressed genes 75 % were found to have two-fold or greater changes in expression as a function of passage based on the microarray analysis (one-way ANOVA *P* value ≤0.05, n = 3). Shown are the log_2_ transformed P5:P0 expression ratios for genes whose association has been replicated in multiple studies or identified in recent large cohort genome wide association or single loci analysis. The corresponding citations (Ref.) are indicated on the right

In addition to evaluating the overlap between genes with expression changes in the wound response model and in AMD, genes that have been identified as risk factors in AMD were evaluated. Fig. 9 shows the expression data for a number of AMD-risk genes with two-fold or greater differences as a function of passage. In large part, the analysis focused on genes whose association has been corroborated in multiple studies or large cohort genome-wide association studies [58–97].

The majority of AMD genetic risk is attributed to variants of several components of the complement cascade; of which *CFH*, *CFB*, *CFI*, *C2*, and *C3* are differentially expressed in the wound response model. However, the net effect of the observed changes on complement activity is unclear. The reduced expression of *CFH* in subconfluent early-passage cells (3 days, P0) and reduced expression of *CFI* in late-passage confluent cells (16–64 days, P5) cells should act to promote complement activity, whereas decreased expression of *CFB*, *C2*, and *C3* should lead to a lowered complement response. Expression of the copy number variant associated complement genes, *CFHR1* and *CFHR3* was not detected in any RPE cell culture. Expression of *HTRA1*, which is located at the other major AMD loci, is also substantially altered with four-fold higher levels being found in confluent late-passage RPE. Consistent with the potential role of wound response in advanced AMD, experimentally induced HTRA1 expression in RPE results in altered Brüch’s membrane structure, polypoidal choroidal vasculopathy, and degeneration of the RPE and photoreceptors in mouse models [98, 99]. However, the basis of these experimental outcomes in the context of TGFβ mediated wound response is unclear as HTRA1 has also been shown to inhibit TGFβ signaling through proteolysis of the ligands and receptors [100–103]. The expression profile of *ARMS2*, which is in linkage disequilibrium with *HTRA1*, could not be evaluated due to lack of representation on the microarray.

In the context of TGFβ mediated wound responses, the AMD-associated genes *TGFBR1* and *GDF6* are most interesting. TGFBR1, whose expression increases post-confluence as a function of passage, is a target of A-83-01 and primary transducer of TGFB1 and TGFB2 activity. GDF6 is nearly exclusive to P5 RPE cells and as such it is a candidate ligand for mediating the irreversible switch in phenotype following sustained wound stimulus. Although GDF6 is essential for normal eye development and decreased GDF6 expression or function can result in ocular defects, microphthalmia, or anaophthalmia [104], its post-development roles are uncertain and there is precedence for TGFβ ligands having opposite effects in the regulation of development and stasis. For example, activin A (an INHBA dimer), promotes the differentiation of the RPE during development and from stem cells [105, 106] and also promotes wound responses in the adult [107]. Promotion of angiogenesis is a well-known component of wound response and the role of VEGFA, both functionally and genetically in AMD is well established. VEGFA expression is substantially reduced in subconfluent RPE and in confluent P5 RPE relative to differentiated RPE. Although this seems contradictory with the fact that neovascularization is a defining feature of CNV, it has been reported that there is a reduction in the choriocapillaris in the margins surrounding the neovascular lesion [108]. A similar reduction in the choriocapillaris was also observed in GA. Both FILIP1L and TNFRSF10A are reported to be involved in promoting apoptosis. Consistent with this function, along with the increased cell death observed in highly passaged RPE and in GA, the expression of *FILIP1L* and *TNFRSF10A* is more than four times higher in confluent P5 RPE cells. Finally, there are a number of genes involved in lipid metabolism with genetic variants associated to AMD, including *LPL*, *SCARB1*, *APOE*, *FADS1*, *FADS2*, and *LRP6*. All of these genes are expressed at significantly lower levels in highly passaged RPE. There was no evidence of expression of two other lipid related AMD genes, *LIPC* and *CETP*, under any condition.

## Discussion

In this study we investigated the molecular basis of intrinsic RPE wound repair, how extended periods of wound stimulus lead to a chronic state of wound response that is independent of further stimulus, and how these processes might relate to the progression of AMD. Using a cell culture model system that views disruption of cell monolayers during routine passage as a wound stimulus, we showed that when repeatedly passaged RPE reach confluence they fail to achieve a normal cell density, they fail to inactivate genes associated with successful wound repair, they fail to reactivate genes associated with terminally differentiated RPE, and they aberrantly upregulate genes that are unique to a terminal mesenchymal phenotype. With respect to identifying the mechanisms that account for the change in behavior, we identified a core set of genes with altered expression in subconfluent, repeatedly passaged cells. An analysis of this subset of genes revealed that they can be organized into an interactome comprised of two modules; one consisting primarily of genes involved in cell division and the other containing genes often associated with wound response, development, or stasis. As a rule, the directions of the changes in expression are consistent with a passage-dependent decrease in mitosis and activation of a wound repair program.

Using pharmacological experiments, we found that stimulation of mitosis using FGF2 or repression of wound responses using TGFBR1/ACVR1B kinase or Rho-associated, coiled-coil containing kinase inhibitors all promoted RPE differentiation at plating densities lower than normally required for the establishment of the RPE phenotype. In addition, when RPE cells were passaged in the presence of the TGFβ receptor inhibitor, A-83-01, there was a substantial increase in the number of times cells could be passaged and retain the capacity to acquire a pigmented epithelial morphology. More strikingly, when cells were passaged in normal medium and subsequently treated with A-83-01, they regained the capacity to regenerate the characteristic pigmented epithelial morphology. In addition, the finding that sustained inhibition of TGFβ signaling does not prevent normal RPE differentiation demonstrates that TGFβ signaling is not essential for RPE differentiation or productive RPE wound repair.

A major objective of this study was to identify the regulatory mechanisms that account for the change in terminal phenotype following protracted subconfluent culture. In that regard, a link between decreased rates of cell division and loss of capacity to establish an epithelial phenotype is not inherently apparent. Both early- and late-passage cells achieve confluence and the cell density at the point of confluence appears roughly equivalent. A potentially significant growth-related difference is that differentiation-competent cells attain a higher final cell density; the implication being that they continue to divide for a period of time post-confluence. The passage dependent changes in the expression of multiple cyclin-dependent kinase inhibitors may be important in this respect. Most notable, are CDKN2A and CDKN2B which are expressed at substantially higher levels in late-passage cells and CDKN1C which is preferentially expressed in early-passage cells. TGFβ induced increases in CDKN2B have been linked to decreased cell proliferation in multiple cell types, including RPE [109–111]. No passage dependent differences in the expression of FGF family ligands or receptors in subconfluent cells were observed, suggesting that changes in FGF signaling are not responsible for the changes in phenotype. It is possible, that continuation of cell division post-confluence may be essential for establishing an ordered epithelial monolayer, establishment of apical-basal polarity, and the subsequent induction of RPE specific gene expression.

ROCK is best known for its role in regulation of the actin cytoskeleton, but it has many additional targets and has been linked to multiple functions including cell motility, establishment of cell polarity, cell proliferation, and cell death [112–114]. ROCK1 and ROCK2 participate in multiple pathways including the TGFβ, WNT, cadherin, and integrin pathways. Moreover, inhibition of ROCK signaling has been shown to mitigate experimentally induced EMT in a number cell types as well as RPE [115–121]. Given the widespread involvement of ROCK in EMT it is not unexpected that inhibition of ROCK would aid in the preservation of the RPE phenotype. However, due to ROCK’s promiscuous involvement in several pathways little certainty is gained regarding the regulatory signals that lead to an epithelial-to-mesenchymal switch. Despite this lack of mechanistic clarity, it is worth noting that we have shown that the ROCK inhibitor Y-27632 also extends the number of passages that stem cell derived RPE can undergo and maintain their normal phenotype and function [122]. We have observed similar effects of ROCK inhibition on preventing the passage-dependent loss of RPE phenotype using fetal RPE and thiazovivin, although the effect may be less efficacious than TGFBR1/ACRV1B inhibition with respect to reversal of the effects of passage or low density plating (M. Radeke and R. Croze, unpublished observations).

The role of altered TGFβ family signaling in the passage-dependent switch to a mesenchymal phenotype is clearly demonstrated by the ability of the TGFBR1/ACVR1B kinase inhibitors to block and reverse this phenomenon. The TGFβ superfamily is comprised of over 30 different ligands; TGFβs, activins and inhibins, bone morphogenesis proteins (BMP), growth differentiation factors (GDF), and nodal. While the ligands can be categorized based on sequence homology, they may be best segregated based on whether they signal via receptors that employ SMAD2/3 or SMAD1/5/8 in their signal transduction pathways. Because TGFβs predominantly utilize SMAD2/3 and BMPs generally act via SMAD1/5/8 these homologous pathways are often referred to as the TGFβ and BMP pathways. Depending on context, activins and some GDFs can use either or both pathways. The TGFβ pathway is often assigned to directing mesenchymal differentiation and maintenance of pluripotency, while the BMP pathway is ascribed to promoting the differentiation of various cell types. In addition to the canonical SMAD-dependent pathway, signaling can occur through the enlistment of multiple non-canonical pathways employing RHOA/ROCK, TAB1/TAK1, PI3K, PP2A, or RAS [123].

Analysis of the passage-dependent changes in expression of 114 genes associated with the TGFβ and BMP pathways revealed that over half of the genes had significant passage-dependent changes in expression of two-fold or greater. However when the analysis is limited to those genes with altered expression in subconfluent cells, only the ligands TGFB1,TGFB2, INHBA, INHBB, and GDF6 and the ligand interactors THBS1, CTGF, and DCN stand out after considering the direction of change and possible mode of action. The synthesis of TGFβ by RPE and its enhanced production following wounding [124–126], as well as its effect on proliferation and promotion of mesenchymal phenotypic changes in RPE [39, 127–129] have been recognized for some time. Similarly, INHBA has been shown to be expressed by RPE and, like TGFβ it inhibits RPE proliferation [130]. It is tempting to speculate whether these differences in expression of the TGFβ and activin ligands account for the marked change in behavior following sustained subconfluent culture. It is not clear, however, that these changes in gene expression are sufficient to cause the marked change in post-confluence phenotype, since both minimally and highly passage cells express significant levels of these genes prior to establishing a confluent monolayer. From this perspective the expression profile of GDF6 is intriguing. There is little to no expression in early-passage cells and there is more than a ten-fold increase in highly passaged cells irrespective of state of confluence. While current knowledge on GDF6 is limited, there is evidence suggesting a possible role in EMT. GDF6 can promote differentiation of cells of mesenchymal origin, and it can bind to the type 2 receptor, ACVR2B, with high affinity [131–133]. ACVR2B is known to associate with ACVR1B (ALK4), an activator of SMAD2/3 [134]. Whether GDF6 can signal through ACRV1B is uncertain as the only cell type where GDF6 signal transduction has been studied does not express ACRV1B and *in vitro* analysis has failed to detect ACRV1B binding. Based on our analysis, cultured RPE cells have substantial levels of ACRV1B expression, irrespective of passage or time.

Assuming that the changes in gene expression result in increased protein expression, it is possible that the ligands may not be available in a biologically active form. Both INHBA and INHBB are inhibited by follistatin (FST) [135], which is also expressed at elevated levels in late-passage cells. TGFB1 and TGFB2 are secreted as latent complexes with the TGFβ binding proteins, LTBP1 and LTBP2 [136, 137], whose expression also increases with passage. In this context the elevated expression of THBS1, CTGF, and DCN are most interesting. In addition to their role in latent TGFβ activation or potentiation, it has been shown that THBS1 treatment of RPE leads to TGFB1 activation [138] and CTGF treatment of RPE stimulates migration and increases the expression of cellular fibronectin, a marker of EMT [139, 140]. Like GDF6, DCN is highly upregulated in both subconfluent and confluent late-passage cells and there is little to no expression in early-passage cells. In addition to promoting the action of TGFβs in proliferating myoblasts [52–55], DCN can cause increased expression and secretion of THSB1 [141, 142]. Taken together, the passage-dependent increase in expression of multiple TGFβ ligands and enhancers of TGFβ signaling could provide a potent mechanism for sustained activation of the TGFβ pathway.

While TGFβ mediated-EMT plays a dominant role in the loss of RPE phenotype with increasing passage, our results suggest that there are other passage-dependent processes that contribute to loss of capacity to differentiate. Even in the presence of sustained repression of mesenchymal gene expression by A-83-01, there is a steady decrease in a subset of RPE-specific genes and an eventual loss of RPE phenotype with extensive passage. The identity of these other processes remains unknown. Clues as to their identity might be found in the small subset of passage-dependent genes that do not respond to TGFβ pathway inhibition, although that will likely require determination of the optimal small molecule inhibitor, doses, and treatment schedules in order to minimize confounding effects due to off-target drug effects. The ultimate loss of phenotype appears not to be related to terminal senescence as P7 cells are still mitotic and largely negative (>85 %) for senescence-associated β-galactosidase (unpublished observations).

Consistent with the hypothesis that the transition from early AMD to advanced AMD is associated with the onset of a dysregulated RPE wound response, we found an overrepresentation of genes whose expression changes after prolonged subconfluent culture among the genes with differential expression in CNV and GA RPE-choroid and retina. In the RPE-choroid there was evidence of wound response in both macular and extramacular regions, whereas in the retina the region of response was limited to the macula, as is the primary visual loss. In the CNV RPE-choroid more than 35 % of the overlapping genes are overexpressed in subconfluent late-passage RPE cells. Among this list are *TGFB2*, *CTGF*, *DCN*, *LTBP1*, and *CDKN2A*; as well as *SPP1*, the most highly overexpressed gene in 3-day late-passage cells. *SPP1*, is known to be upregulated by TGFβ in bone cells and it is essential for TGFB1 induced cardiac myofibroblast differentiation [143–145]. This differs with the situation in GA where less than 20 % of the overlapping genes are overexpressed in subconfluent late-passage cells. Moreover, there is essentially no representation of the TGFβ pathway genes in the RPE-choroid GA data set. This difference might reflect the fact that in CNV a majority of the cells in the neovascular lesions are TGFβ-immunoreactive RPE and fibroblast-like cells of possible RPE origin [146, 147]. In contrast, in GA the disease state is best defined by cell death, with the actively involved cells possibly being limited to the margins of the disease area. In the retina the one differentially expressed gene of note in the context of this study is TGFB2, which is overexpressed in both CNV and GA. This evidence of the association of a RPE wound response in advanced AMD, coupled with prior reports of elevated TGFβ expression in AMD and linkage of a *TGFBR1* polymorphism with AMD risk all point to dysregulation of TGFβ signaling as a contributing factor in AMD progression.

Finally, when contrasting a culture based system with *in vivo* disease one must keep in mind the differences between the two cases. In the tissue culture model there is a single cell type and a clearly defined stimulus. Whereas in the disease, there are multiple cell types and processes that contribute to the disease and can muddy analysis. In addition, there are aspects of the model system that may not truly reflect the disease state or process. Some of these differences may account for the lack of evidence of wound responses in early AMD. Also, in the early stages of AMD the affected areas are minimal with respect to the tissue as a whole and variable between donors; the net result being a dilution of signals and increased noise due to inter-patient variability. Determining the extent to which wound responses might be involved in early AMD and the transition to advanced dry AMD will likely require analysis of RPE cells in the direct proximity of drusen and the margins of geographic atrophy.

## Conclusions

In the broadest sense, wound responses encompass the cellular reactions to disruption of normal tissue or cellular architecture. By this definition, wound responses are inherent components of degenerative disease. Here, we have shown that when RPE cells are subjected to sustained wound stimulus by impairing the establishment of cell-cell contacts, there is a self-perpetuating activation of TGFβ pathway that persists even after a confluent monolayer is reestablished. The net result is permanent loss of RPE function and establishment of a chronic state of wound response. Moreover, we show that there is significant overlap among the genes whose expression change in the RPE wound response model and the genes with altered expression in advanced AMD. Currently, the only therapeutic for the treatment of AMD is directed at the inhibition of neovascularization using function blocking anti-VEGF antibodies. However, this treatment does not stop the progression of the non-vascular aspects of the disease [148]. An approach directed at preventing misappropriate activation of TGFβ signaling might inhibit the advancement of AMD in general. In addition, while the focus here has been on the role of wound response in AMD, these findings are likely to be relevant to other diseases involving the RPE, such as proliferative vitreoretinopathy, and epithelial wound responses in general. Moreover, the potential to extend the effective lifespan of RPE and perhaps other epithelial cell types in culture through the use of TGFβ receptor inhibitors is of clear practical impact with respect to the generation of copious quantities of primary epithelial cells for research or cell-based therapeutics. In the future it will be interesting to determine the direct effects of TGFβ ligands on differentiated RPE monolayers and the potential interplay with other aspects of AMD such as oxidative stress and inflammation.

## Additional files

Additional file 1: Table S1. List of real-time quantitative PCR assays, primers and probes. Additional file 2: Figures S1 to S7. Supplemental figures and legends. Additional file 3: Table S2. Accompanying data for the cluster analysis depicted in Fig. 2.

## Abbreviations

AMD: Age-related macular degeneration
CNV: Choroidal neovascularization
EMT: Epithelial-to-mesenchymal transitions
GA: Geographic atrophy
P: Passage
RT-qPCR: Real-time quantitative PCR
RPE: Retinal pigmented epithelial. Genes, RNAs, and proteins are all abbreviated using the current HUGO Gene Nomenclature Committee symbols
