# Supplementary material for: Restoration of mesenchymal retinal pigmented epithelial cells by TGFβ pathway inhibitors: implications for age-related macular degeneration
Source: Genome Med. 2015 Jun 19;7(1):58. doi: 10.1186/s13073-015-0183-x (PMC4491894; doi:10.1186/s13073-015-0183-x)
Supplement: Additional file 2: Figures S1 to S7. — Supplemental figures and legends. [file 13073_2015_183_MOESM2_ESM.docx]

**Restoration of mesenchymal retinal pigmented epithelial cells by TGFβ pathway inhibitors: implications for age-related macular degeneration**

Monte J. Radeke^1^, Carolyn M. Radeke^1^, Ying-Hsuan Shih^1^, Jane Hu^2^, Dean Bok^2^, Lincoln V. Johnson^1^ and Pete J. Coffey^1^

^1^Neuroscience Research Institute

University of California, Santa Barbara

^2^Departments of Ophthalmology and Neurobiology

Jules Stein Eye & Brain Research Institutes

David Geffen School of Medicine

University of California, Los Angeles

**Supplemental Figures 1 - 7**


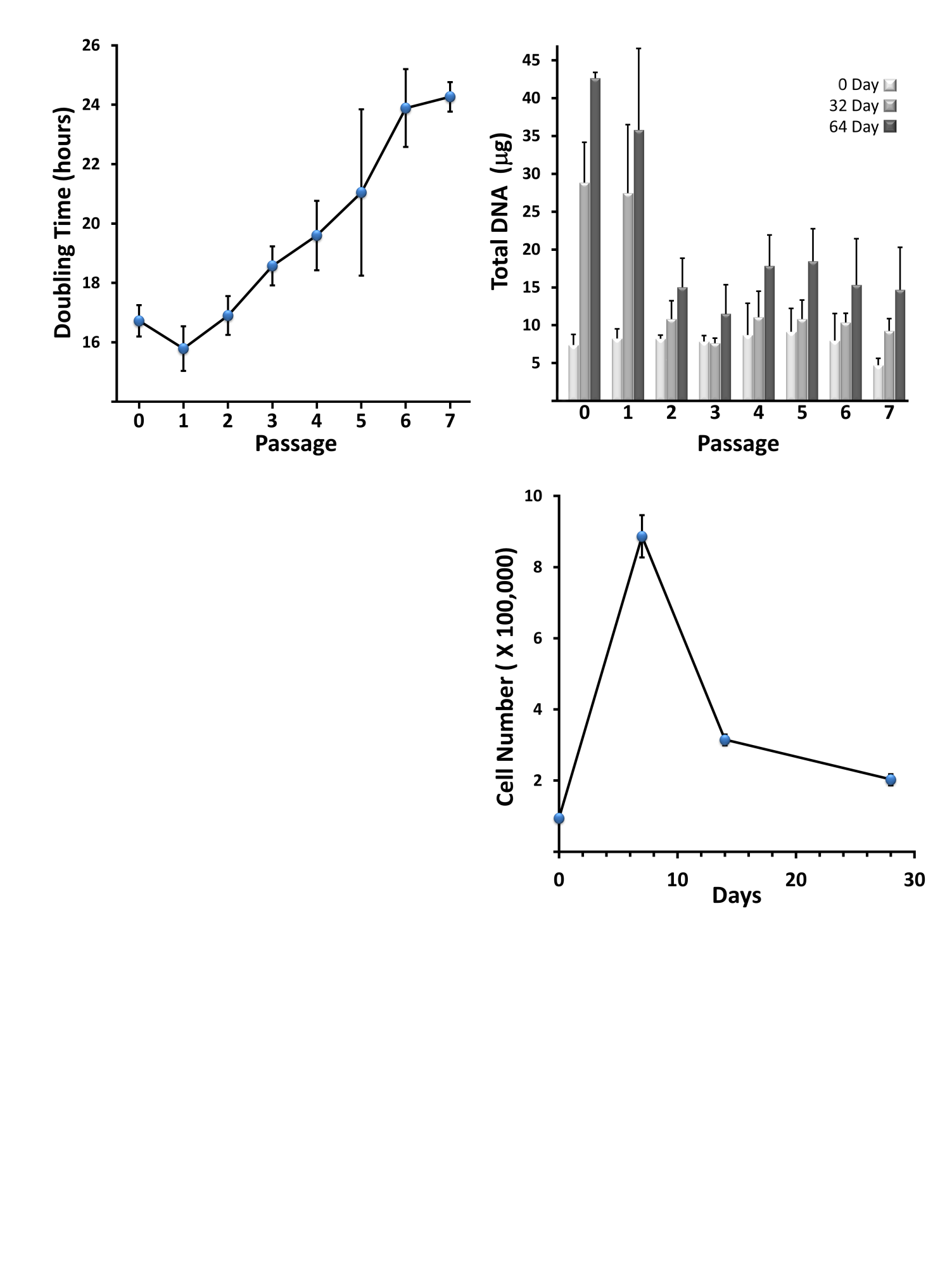


**Fig. S1 Prolonged culture at subconfluence results in decreased rate of cell division and cell density.** (A) There is an increase in doubling time as a function of passage. Cells were serially passaged as described in *Methods*. At each passage the total cell number was determined by cell counting and the doubling time was calculated (n = 3, +/- SEM). (B) Low passage, differentiated RPE cultures have a higher final cell density than highly passaged, non-differentiated cultures. At each passage cells were plated at 80,000 cells/cm^2^. At 0, 32, and 64 days post-plating the cells were harvested and the amount of DNA per culture was determined (3 biological replicates, 2 technical replicates each, +/- SEM). Coincident with the passage at which the cells no longer differentiate (see Fig. 1) there is a sharp decrease in the amount of genomic DNA at confluence. (C) There is a decrease in cell density post-confluence when RPE cells are plated at low density and fail to differentiate. Passage 0 cells were plated at 10,000 cells/cm^2^ and maintained without further passage. Confluence was obtained within 7 days (not shown). At the indicated time the cultures were harvested and the total number of cells was determined by counting (n = 3, +/- SEM).

**A**

**C**

**B**


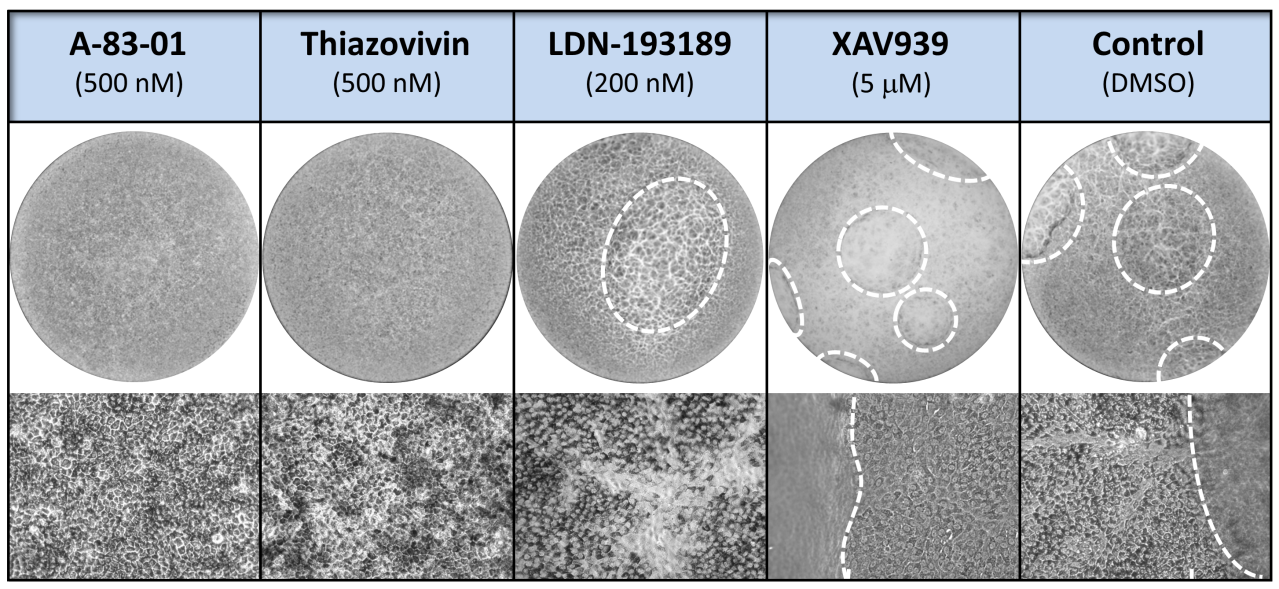


**Fig. S2 The effects of small molecule inhibitors on RPE cell differentiation on minimally passaged high density RPE cells.** Passage 0 cells were plated at 80,000 cells/cm^2^ on tissue culture plastic and maintained in the presence of the indicated small molecules. The vehicle alone (DMSO) served as a control. Shown are whole culture images (above) and phase contrast micrographs (below) taken at 32 days. The dashed lines outline areas of doming, fluid filled spaces between the cell monolayer and the substrate. Both A-83-01 and thiazovivin treated cultures exhibit a more uniform level of pigmentation and cobblestone morphology than the control, whereas XAV939 treatment results in reduced pigmentation and altered morphology. No overt effects of LDN-193189 treatment were apparent.


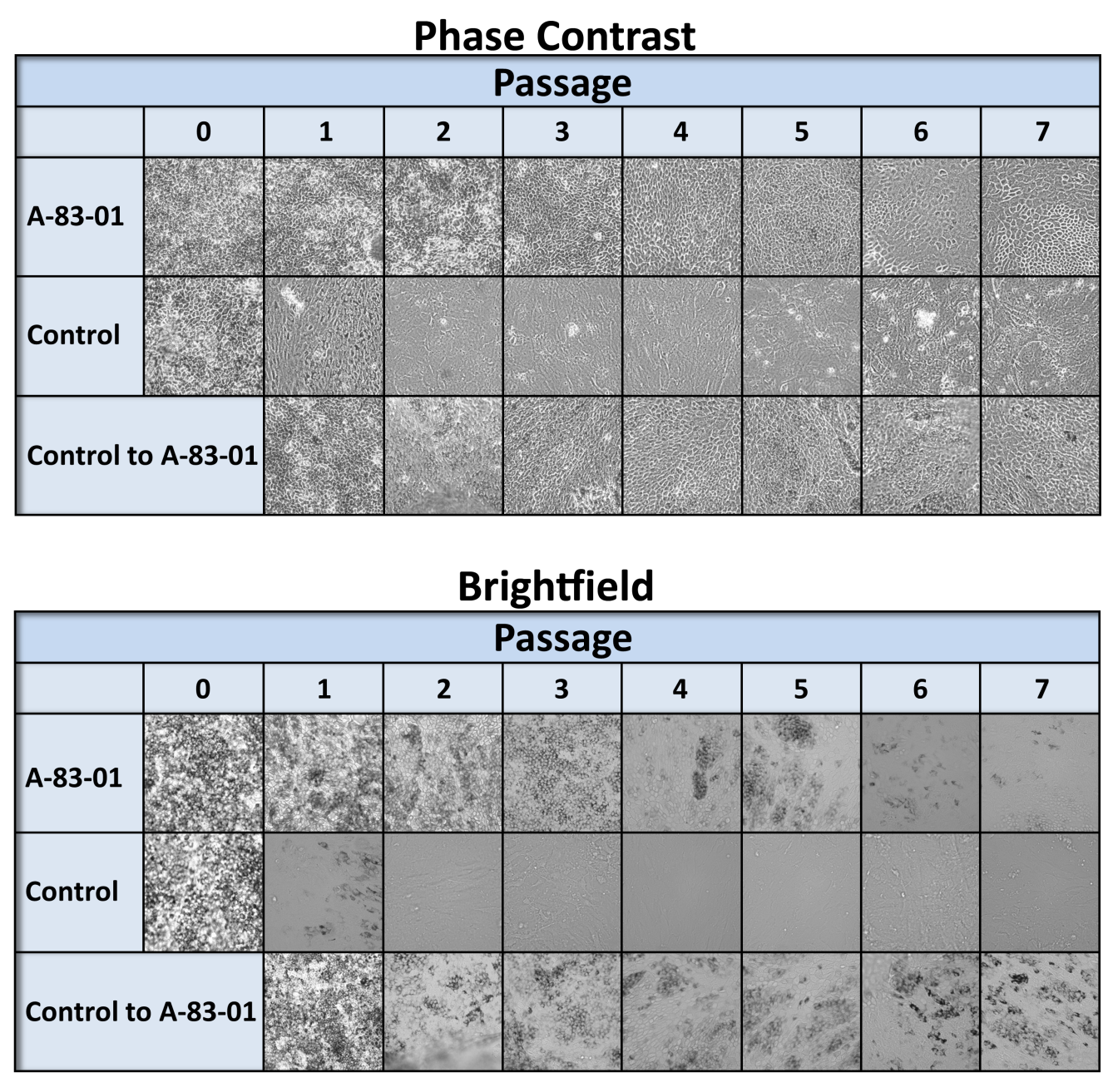


A

**Fig. S3 Inhibition of TGFBR1/ACVR1B signaling prevents and reverses passage dependent loss of epithelial morphology.** (A) Phase contrast and (B) brightfield micrographs of the cultures depicted in Fig. 5. When passaged and maintained in the presence of 500 nM A-83-01, RPE cells retain the capacity to acquire a prototypical cobblestone morphology for at least four passages beyond that of control cultures. Even at P6 and P7 when there is a significant reduction in pigmentation, the overall morphology is markedly improved relative to the control. The same beneficial effects of A-83-01 on cell morphology are even seen when it is added to RPE cells that had undergone multiple passages in normal medium (Control to A-83-01).

**A**

**B**

30 μm


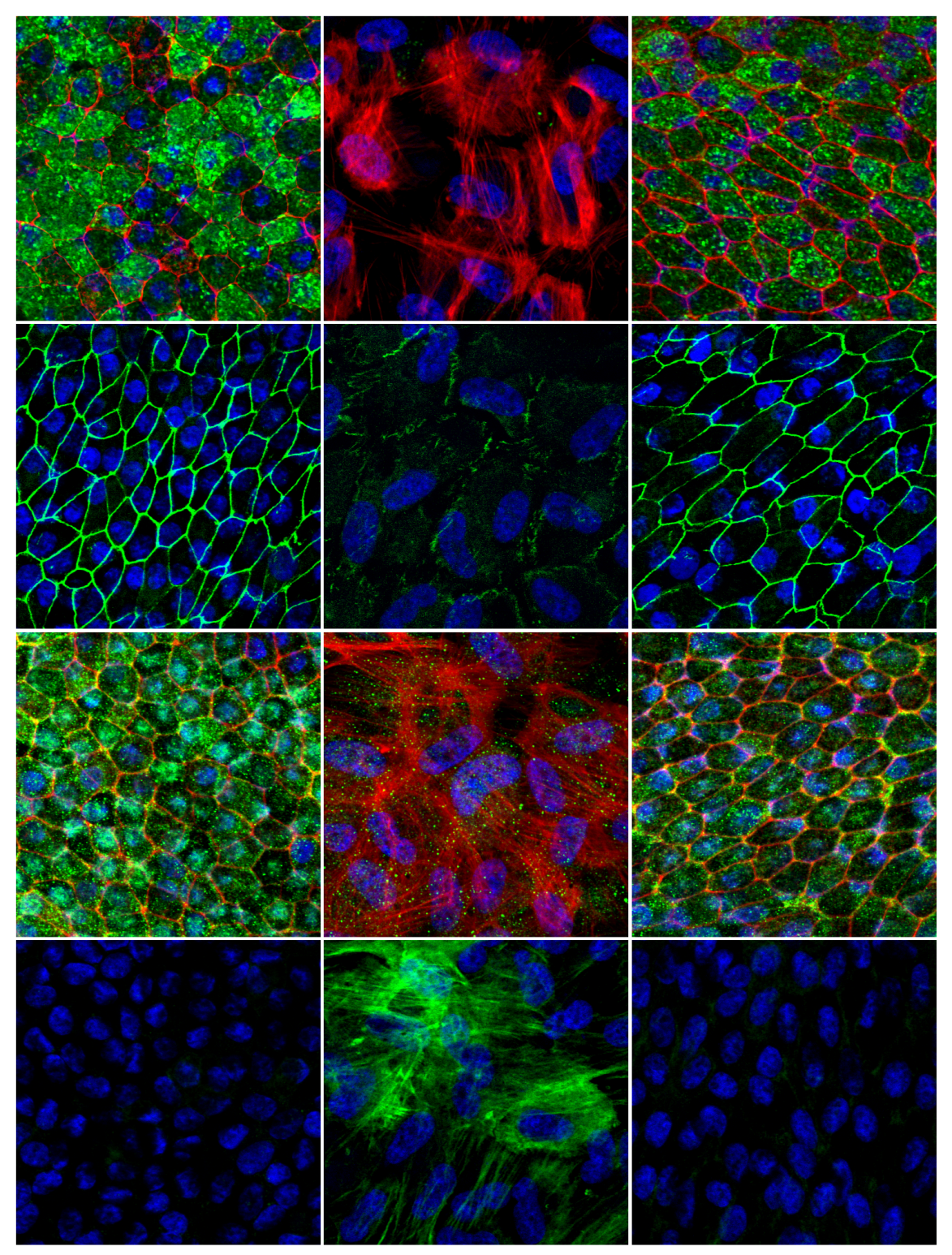


PMEL

Phalloidin

TJP1

BEST1

ACTA2

Phalloidin

Passage 0

Passage 3

Passage 3 + A-83-01

**Fig. S4 A-83-01 treatment preserves the normal expression and subcellular localization of the actin cytoskeleton, PMEL, TJP1, and BEST1.** 32 day cultures of P0 RPE and P4 cells grown in normal medium and P4 cells treated with 50 nM A-83-01 were fixed with paraformaldehyde and the cellular localization of smooth muscle actin (ACTA2), tight junction protein 1 (TJP1; also known as zona occludens 1 or ZO-1), premelanosome protein (PMEL) and bestrophin 1 (BEST1) were determined using immunofluorescence laser scanning confocal microscopy. Antibody labeling is represented by green pseudocoloring. Where indicated, total actin was detected using phalloidin (red). Nuculei were labeled using Hoescht 33342 (blue).

A


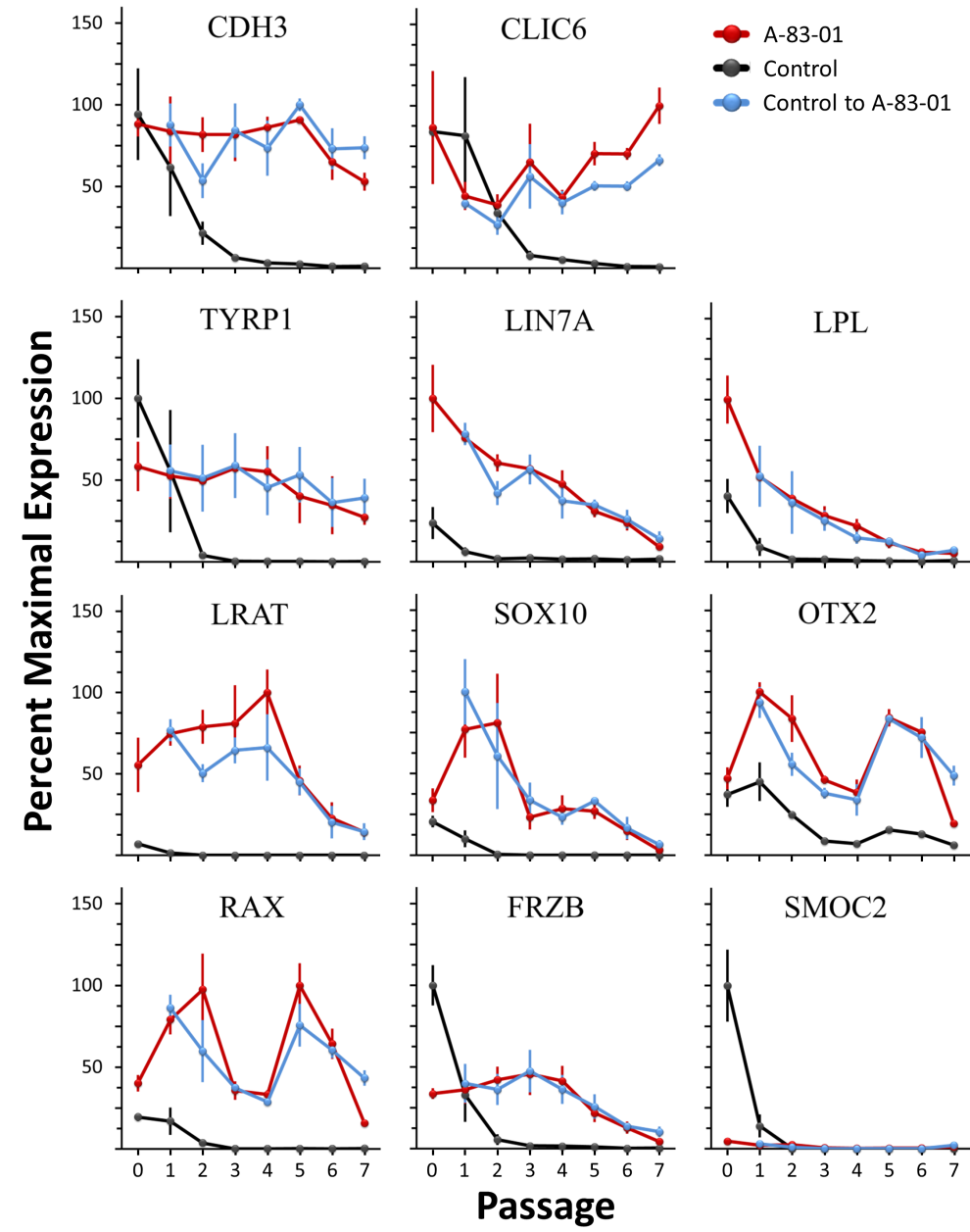


A

**Fig. S5 RPE and Wound Response marker expression profiles as a function of passage and TGFBR1/ACVR1B inhibition.** Shown are the mean expression profiles for cells grown and passaged in the presence (red) or absence (black) of 500 nM A-83-01 or cells grown and passaged in control medium and subsequently transferred to medium containing A-83-01 at the indicated passage number (blue). At each passage an aliquot of cells was plated at 80,000 cells/cm^2^ and maintained without further passage. Relative expression levels of a set of RPE (A) and Wound Response (B) marker genes were determined after 32 days using RT-qPCR (n =3, +/- SEM).


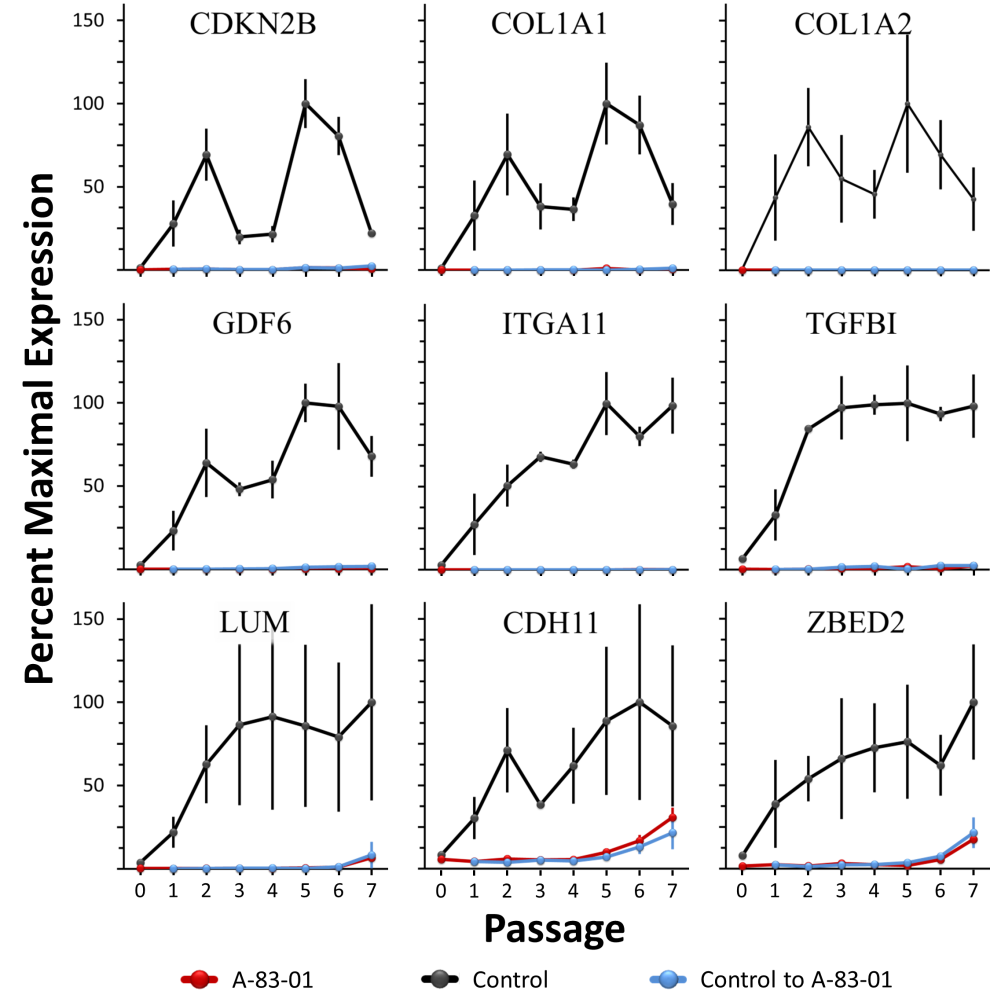


**Fig. S5 (Continued) RPE and Wound Response marker expression profiles as a function of passage and TGFBR1/ACVR1B inhibition.**

B


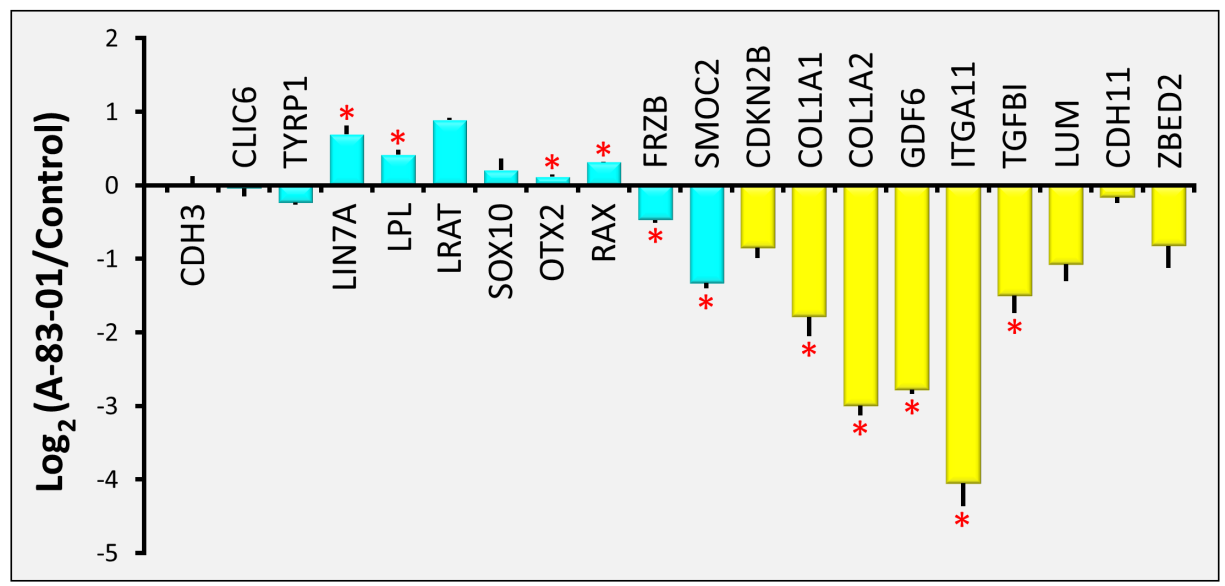


**Fig. S6 A-83-01 treatment results in increased RPE gene expression and decreased wound response genes in minimally passaged, differentiation-competent RPE cells.** Passage 0 cells were plated in normal medium or in medium supplemented with 500 nM A-83-01 at a density of 80,000 cells/cm^2^. After 32 days the level of the genes of interest were determined by RT-qPCR and the log_2_ transformed A-83-01:Control ratios were determined (n = 3, +/- SEM). Values denoted by a red asterisk have *P* values ≤0.05 (paired Student’s t-test). Blue bars indicate RPE genes and yellow bars indicate Wound Response genes.


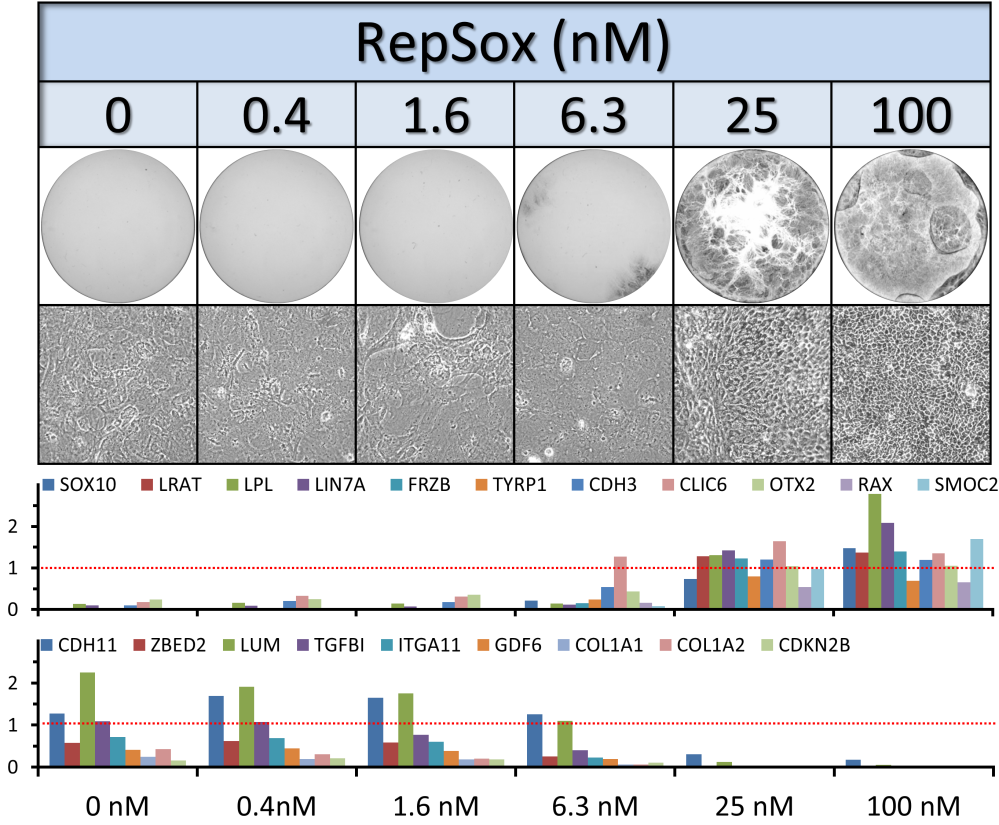

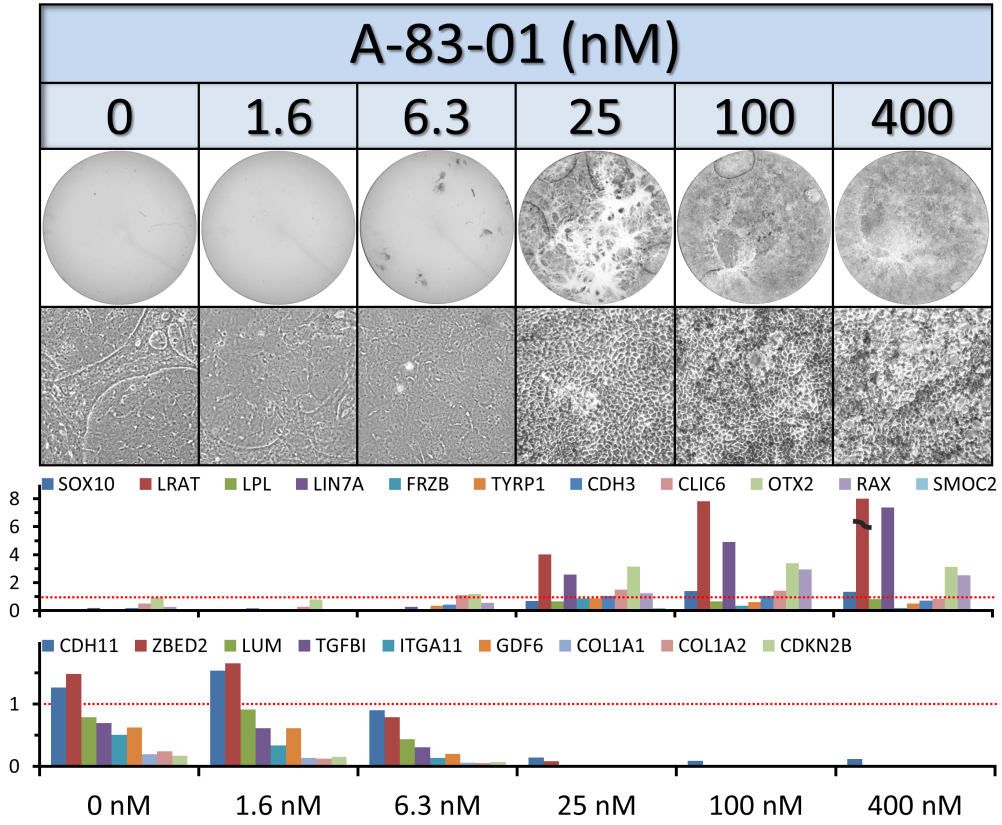


**Fig. S7 TGFBR1/ACVR1B kinase inhibitor dose-response.** Passage 0 cells were plated at 4,000 cells/cm^2^ in medium containing A-83-01 (A), RepSox (B), or SB-431542 (C) at the indicated concentrations. At 32 days, whole culture photographs and phase contrast micrographs were taken to assess the degree of pigmentation and the cell morphology. The effect of the inhibitors on RPE (upper chart) and Wound Response (lower chart) gene expression was determined by comparing the level of expression relative to 32 day differentiated P0 cells (RPE genes) or undifferentiated P5 cells (Wound Response genes) plated at 80,000 cells/cm^2^. The dashed red line highlights the level of equivalency. Note that for many of the wound response genes the level of induction in the low density model is less than that seen in the passage model.

B

A


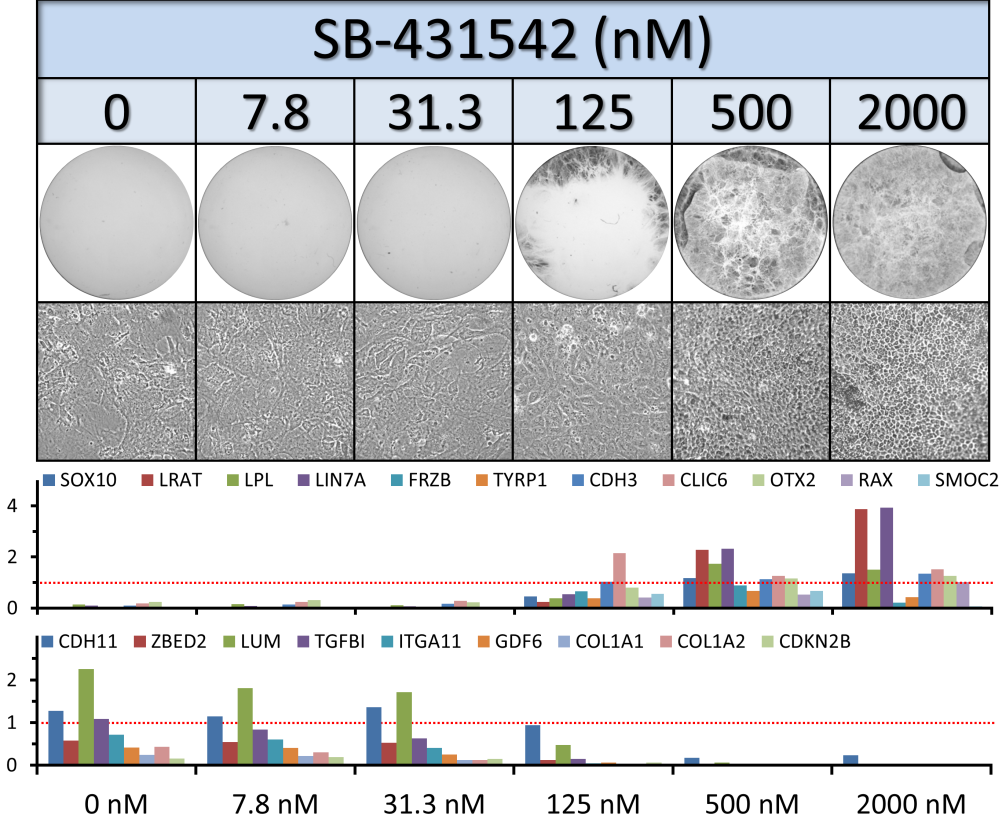


**Fig. S7 (Continued) TGFBR1/ACVR1 kinase inhibitor dose response.**

C
